# Supplementary material for: Dual impact of elevated temperature on plant defence and bacterial virulence in Arabidopsis
Source: Nat Commun. 2017 Nov 27;8:1808. doi: 10.1038/s41467-017-01674-2 (PMC5704021; doi:10.1038/s41467-017-01674-2)
Supplement: Supplementary file 1 — Supplementary Information [file 41467_2017_1674_MOESM1_ESM.pdf]

## Supplementary Figures

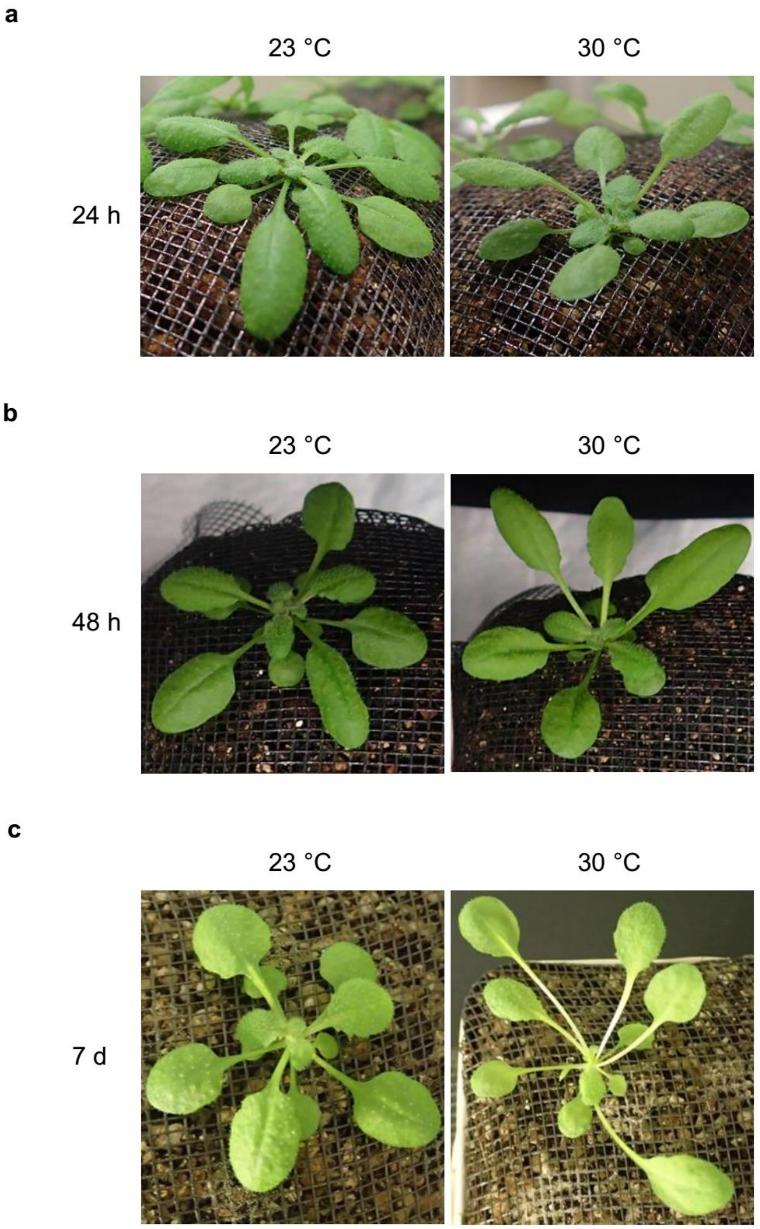


**Supplementary Figure 1. Morphological response of *Arabidopsis* plants to elevated temperature.** Four-week-old plants were shifted to test chambers at 23 °C or 30 °C. Pictures were taken (**a**) 24 h or (**b**) 48 h after temperature shift. (**c**) Two-week-old plants were shifted to test chambers at 23 °C or 30 °C. Pictures were taken 7 days after temperature shift.

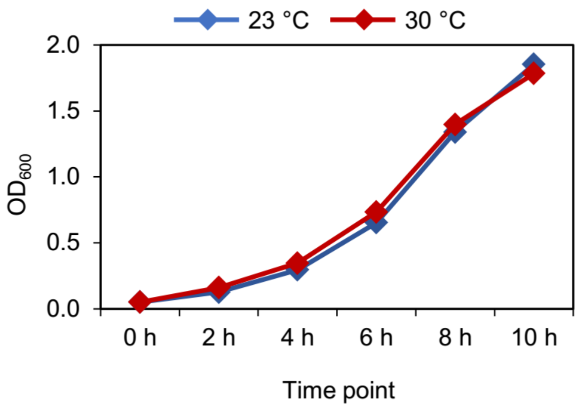


**Supplementary Figure 2. Effect of temperature on *Pst* DC3000 growth *in vitro*.** *Pst* DC3000 was grown in liquid culture at 23 °C or 30 °C. Diamonds indicate the mean reading of three biological replicates ± the standard error of the mean (s.e.m.). Data represent three independent experiments.

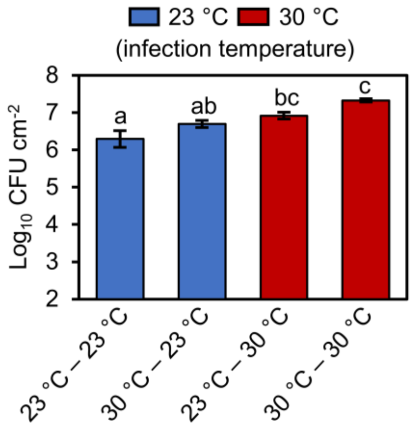


**Supplementary Figure 3. Disease outcomes of *Arabidopsis* plants with indicated temperature treatments before or after infection.** Bacterial growth in plants (n = 4) three days after syringe-infiltration with *Pst* DC3000. Plants were (i) acclimated and kept at 23 °C following infection (23 °C – 23 °C), (ii) acclimated at 30 °C and shifted to 23 °C (30 °C – 23 °C), (iii) acclimated at 23 °C and shifted to 30 °C following infection (23 °C – 30 °C), or (iv) acclimated and kept at 30 °C following infection (30 °C – 30 °C). Data represent three independent experiments, and are presented as the mean ± s.e.m., with n = biological replicates. Letters indicate statistical significance based on a two-factor ANOVA with Tukey's HSD post hoc analysis ($p$-value < 0.05); samples sharing letters are not significantly different.

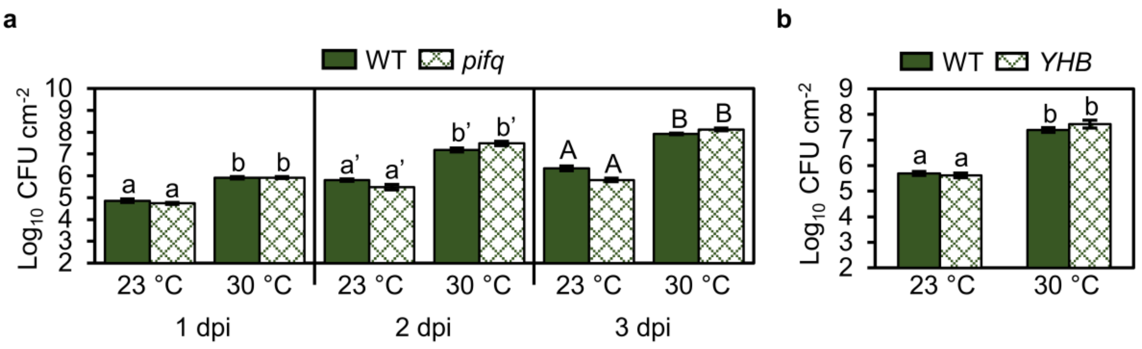


**Supplementary Figure 4. Enhanced growth of *Pst* DC3000 at 30 °C does not require PIFs or phyB.** (**a**) Bacterial growth in WT (Col-0) and *pifq* mutant plants (n = 4) one, two and three days after syringe-infiltration with *Pst* DC3000. (**b**) Bacterial growth in WT (L*er*) and *YHB* transgenic plants (n = 4) three days after syringe-infiltration with *Pst* DC3000. All data are representative of three independent experiments and are presented as the mean ± s.e.m., with n = biological replicates. Letters indicate statistical significance based on a two-factor ANOVA with Tukey's HSD post hoc analysis ($p$-value < 0.05); samples sharing letters are not significantly different. Data for each time point in (a) were analysed separately.

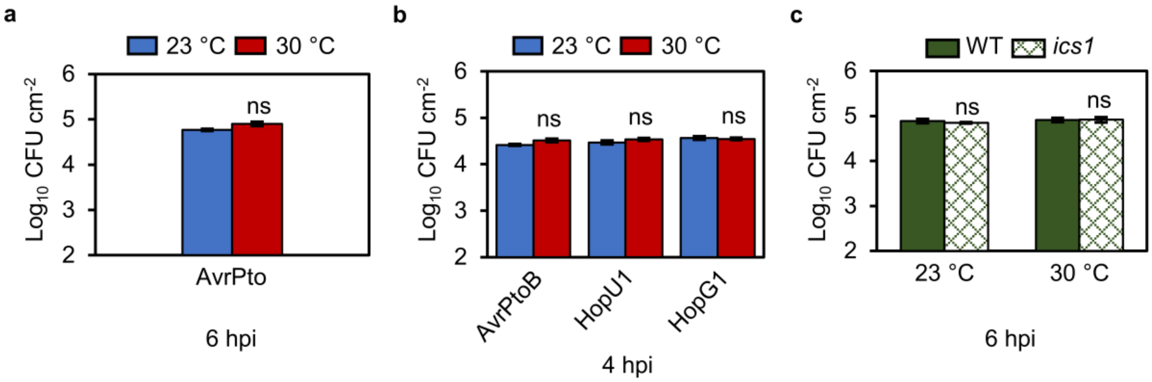


**Supplementary Figure 5. Elevated temperature does not affect bacterial growth *in planta* at 4 to 6 hpi.** Bacterial growth in WT plants (**a**) 6 h after syringe-infiltration with *Pst* DC3000 $P_{nptII}$::*avrPto-CyaA* (n = 4) (**b**) 4 h after syringe-infiltration with *Pst* DC3000($P_{tac}$::*avrPtoB-CyaA*) (n = 4), *Pst* DC3000($P_{tac}$::*HopU1-CyaA*) (n = 6),or *Pst* DC3000($P_{tac}$::*HopG1-CyaA*) (n = 6) strains. (**c**) Bacterial growth in WT and *ics1* mutant plants (n = 4) 6 h after syringe-infiltration with *Pst* DC3000($P_{nptII}$::*avrPto-CyaA*). Data are presented as the mean ± s.e.m. with n = biological replicates, and are representative of three independent experiments. “ns” indicates no statistical significance based on a Student’s *t*-test (*p*-value > 0.05) of pairwise comparisons between samples at 23 °C vs. 30 °C (a and b) or between genotypes at each temperature (c).

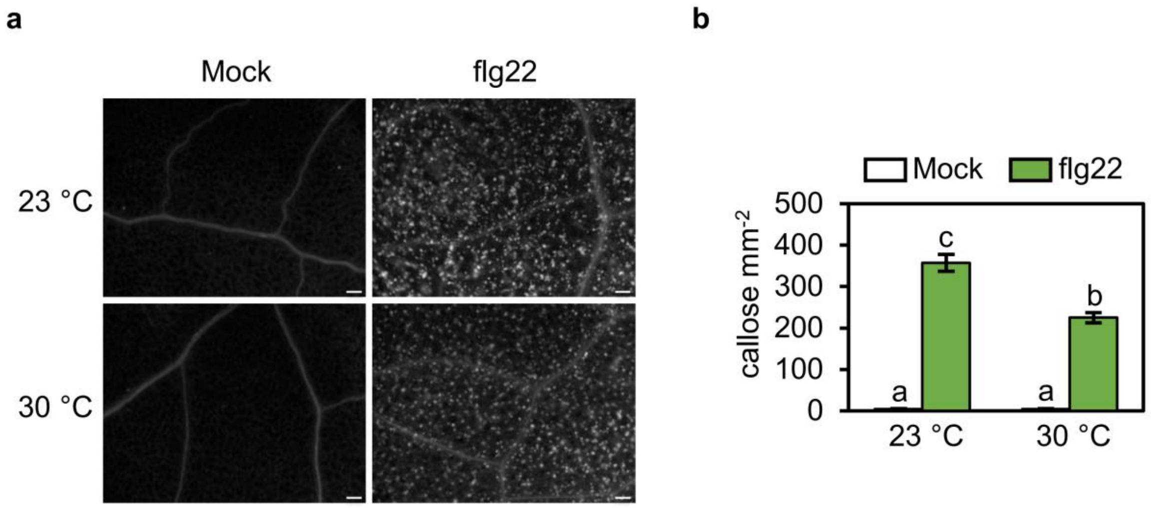


**Supplementary Figure 6. Flg22 treatment induces callose at elevated temperature.** (**a**) Representative images of callose accumulation 24 hpi with mock or flg22 of temperature acclimated plants (n = 6). Scale-bar length represents 100 µm. (**b**) Quantification of callose accumulation from plants treated as described in (a). Data are representative of three independent experiments. Graphical data are presented as the mean ± s.e.m., with n = biological replicates. Letters indicate statistical significance based on a two-factor ANOVA with Tukey's HSD post hoc analysis ($p$-value < 0.05); samples sharing letters are not significantly different.

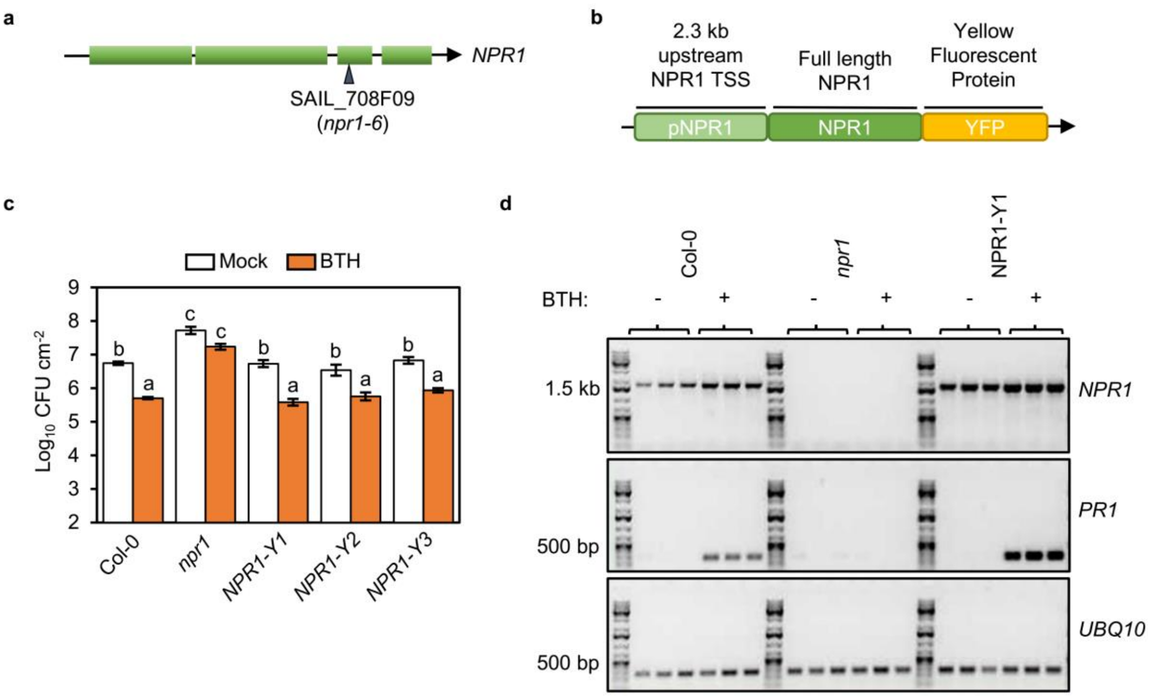


**Supplementary Figure 7. Characterization of *Arabidopsis npr1-6* mutant and *pNPR1::NPR1-YFP* transgenic lines.** (**a**) Model of T-DNA insertion in *NPR1* for the SAIL_708F09 allele, named here *npr1-6*, and referred to as *npr1*. (**b**) Model of *pNPR1::NPR1-YFP* construct. The NPR1 promoter used was 2.3 kb upstream of the NPR1 transcriptional start site (TSS). (**c**) Bacterial growth in five-week-old mock- or BTH-pre-treated plants three days after syringe-infiltration with *Pst* DC3000. Data are presented as the mean (n = 3) ± s.e.m., and are representative of three independent experiments. Letters indicate statistical significance based on a two-factor ANOVA with Tukey's HSD post hoc analysis ($p$-value < 0.05); samples sharing letters are not significantly different. (**d**). Five-week-old plants (n = 3) were sprayed with mock or BTH 24 h prior to harvesting tissue for RNA extraction. Semi-quantitative gene expression analysis was used to determine the expression levels of *NPR1* (35 cycles) and *PR1* (25 cycles) with *UBQ10* (25 cycles) expression used as an internal control. Data are representative of two independent experiments. Primer sequences are provided in Supplementary Table 6.

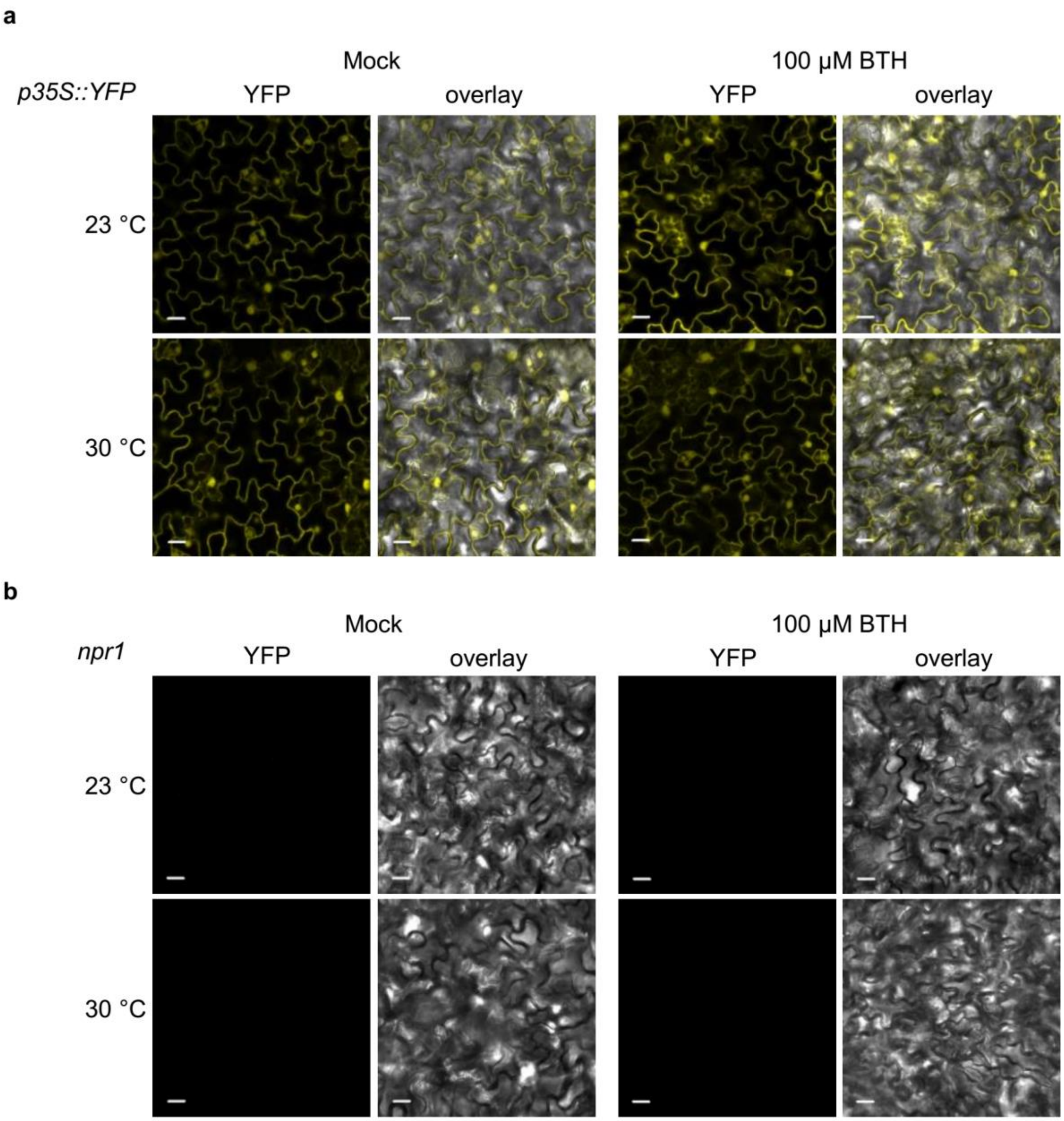


**Supplementary Figure 8. Effect of elevated temperature and BTH on *p35S::YFP* and *npr1-6*.** Representative confocal microscopy images of (**a**) *p35S::YFP* and (**b**) *npr1* plants 24 h after spraying with mock or BTH. Images are of YFP (yellow) alone or YFP overlaid on Brightfield (grey-scale). Scale bar length represents 10 µm. Data are representative of three independent experiments with four biological replicates per experiment.

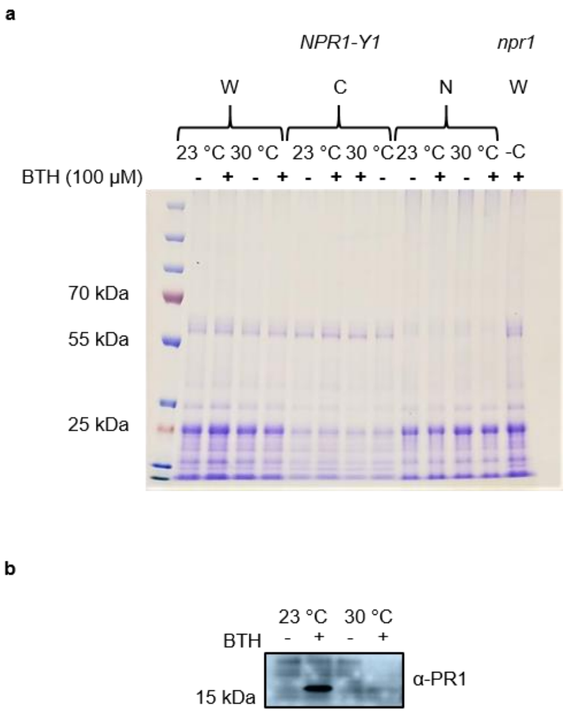


**Supplementary Figure 9. SDS polyacrylamide gel electrophoresis (PAGE) and western blot of leaf proteins.** (**a**) Leaf protein samples of whole cell lysate (W), non-nuclear (C, cytosolic) and nuclear (N) enriched fractions isolated from *NPR1-Y1* transgenic plants treated with mock (-) or BTH (+) solutions at 23 °C or 30 °C were loaded in equal volumes (10 µl) and run in a 4 – 12 % gradient SDS-PAGE gel. Whole cell lysate extracted from *npr1* plants treated with BTH at 23 °C was used as the negative control for the NPR1-YFP band (for western blotting shown in Fig. 4b). The gel was then stained with Coomassie to visualize protein bands for assessment of equal loading. (**b**) Western blot of non-nuclear fraction isolated from leaves pooled from four, temperature acclimated *NPR1-Y1* plants treated with mock (-) or BTH (+). Equal volumes (10 µl) of each protein sample were loaded and run a 4 – 12 % gradient SDS-PAGE gel. Following transfer, the PVDF membrane was probed using a α-PR1 primary antibody (expected MW ~16 kD). Data are representative of three independent experiments.

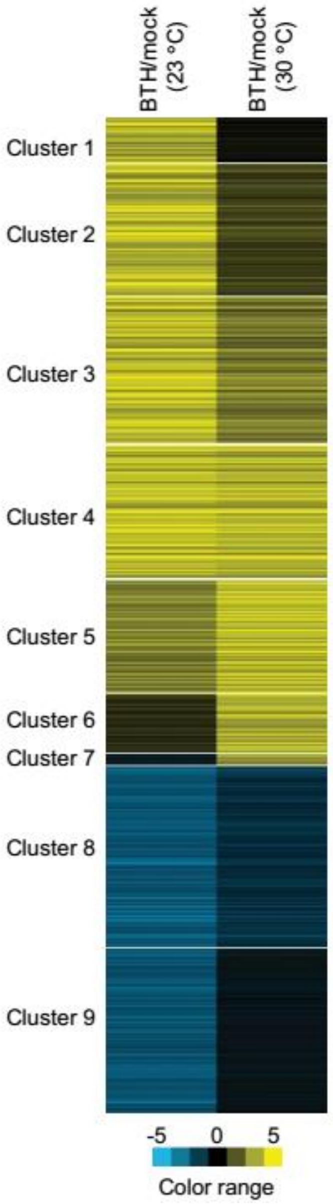


**Supplementary Figure 10. Effect of elevated temperature on the BTH regulated transcriptome.** Differentially expressed genes were identified from RNA-seq data and k-means clustering was conducted as described in Supplementary Note 1 and the main text. The heat map shows a visual representation of gene expression patterns based on fold change (BTH/mock) within each cluster. Down-regulation of expression between the mock- and BTH-treated plants at each temperature is denoted by the level of blue color, up-regulation by the level of yellow color and no change by black color as indicated by the color scale.

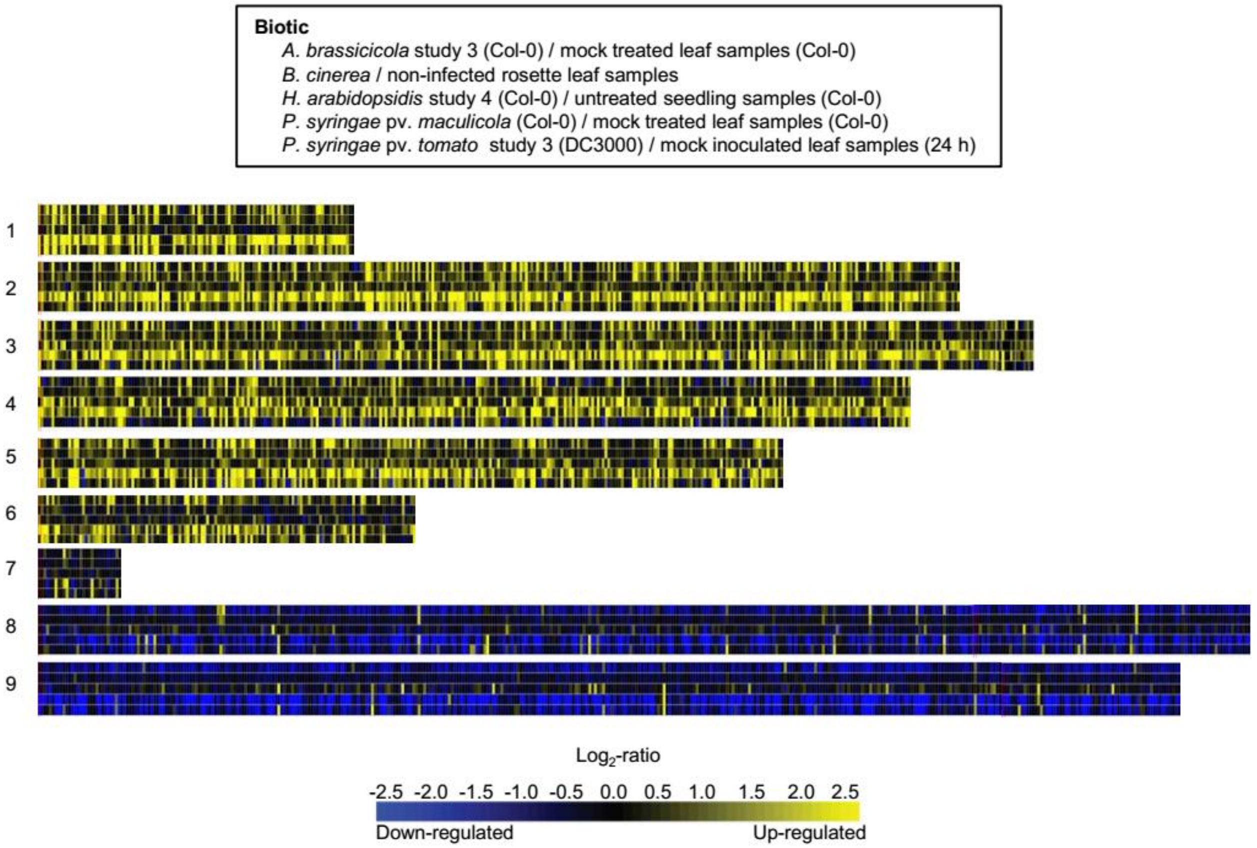


**Supplementary Figure 11. Genevestigator analysis using publicly available microarray expression data of genes in response to biotic stress.** Of the pathogens listed under biotic stress, *Alternaria brassicicola* and *Botrytis cinerea* are necrotrophic pathogens, *Hyaloperonospora arabidopsidis* is an obligate biotrophic pathogen and both strains of *Pseudomonas syringae* are hemi-biotrophic pathogens. Numbers to the left of each panel indicate the cluster to which that set of genes is assigned. Expression values are $\log_2$ ratios of treated vs. untreated or mock controls, with down-regulation of expression denoted by the level of blue color, up-regulated denoted by the level of yellow color and no change in expression denoted by black color as indicated by the color scale.

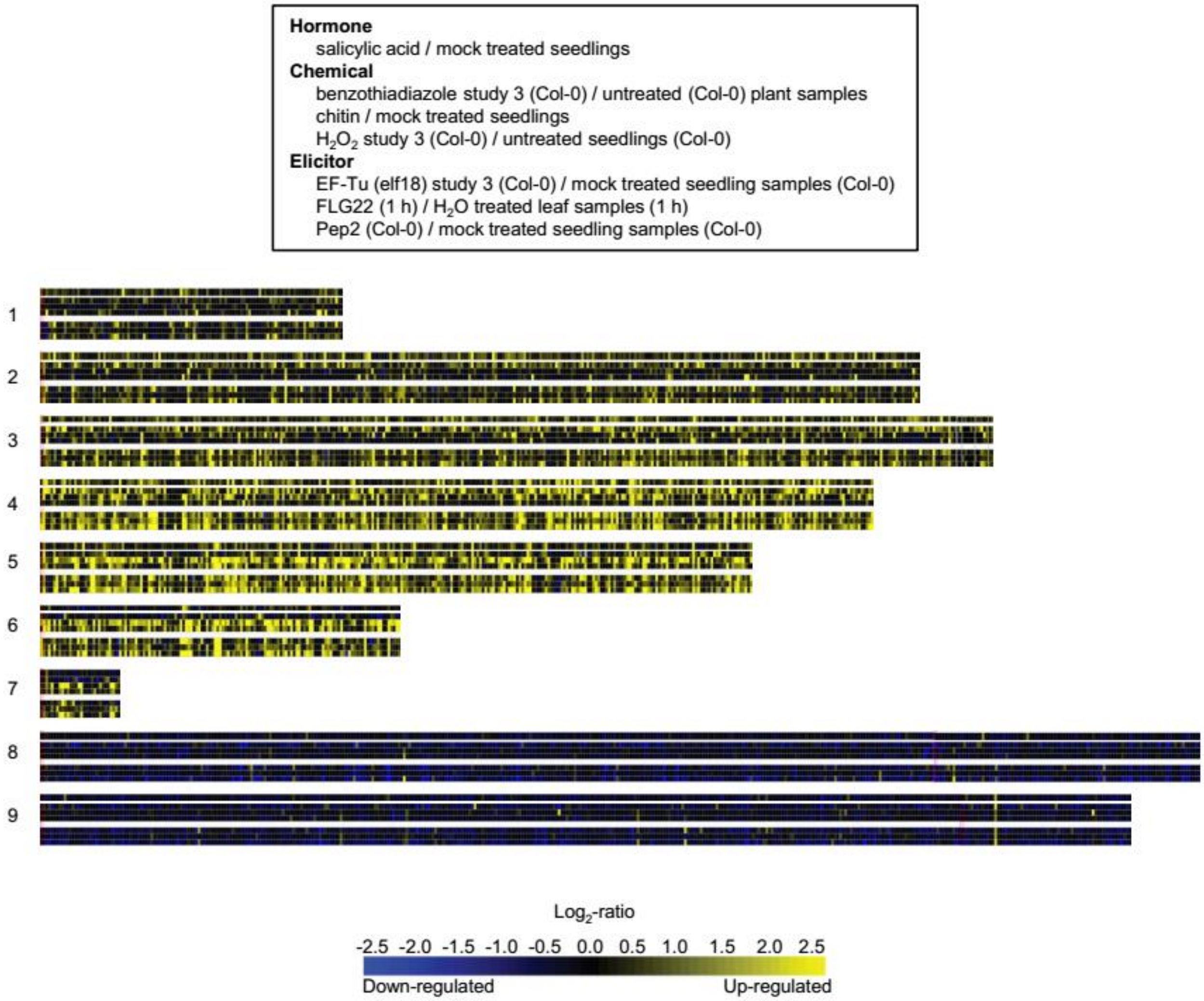


**Supplementary Figure 12. Genevestigator analysis using publicly available microarray expression data genes in response to SA or PAMP elicitors.** Numbers to the left of each panel indicate the cluster to which that set of genes is assigned. Expression values are $\log_2$ ratios of treated vs untreated or mock controls, with down-regulation of expression denoted by the level of blue color, up-regulated denoted by the level of yellow color and no change in expression denoted by black color as indicated by the color scale.

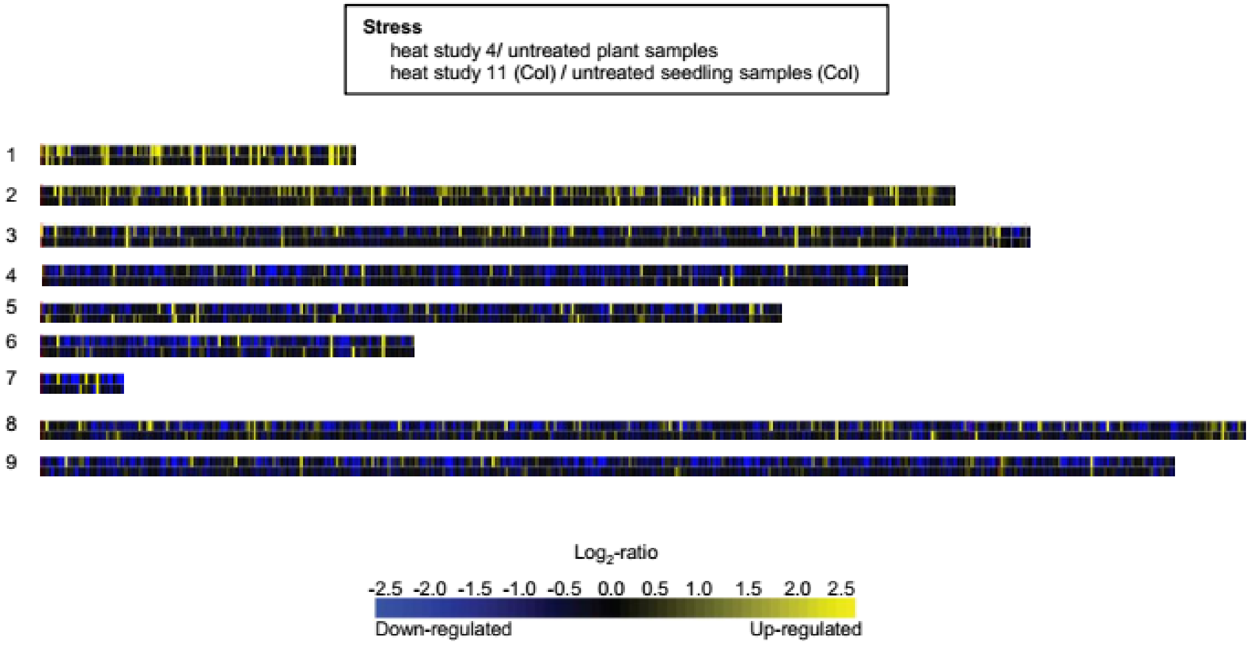


**Supplementary Figure 13. Genevestigator analysis using publicly available microarray expression data of genes in response to heat stress.** Numbers to the left of each panel indicate the cluster to which that set of genes is assigned. Expression values are $log_2$ ratios of treated vs untreated or mock controls, with down-regulation of expression denoted by the level of blue color, up-regulated denoted by the level of yellow color and no change in expression denoted by black color as indicated by the color scale.

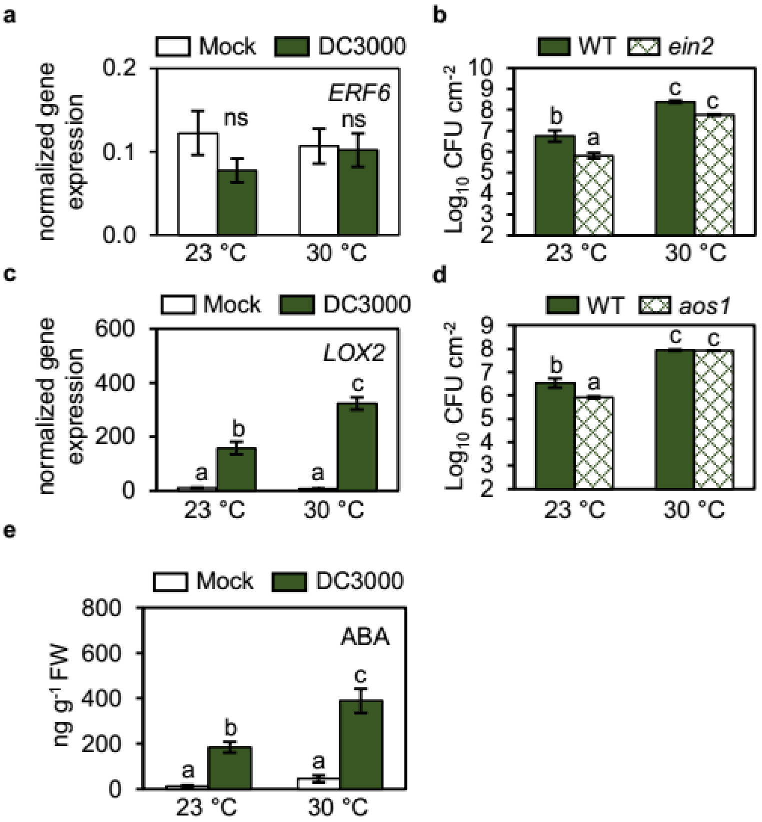


**Supplementary Figure 14. Hormone pathways known to antagonize SA are not involved in elevated temperature-associated enhanced susceptibility.** (**a**) ET and (**c**) JA marker gene expression (n = 3) in plants 24 h after vacuum-infiltration with mock or *Pst* DC3000. qPCR was used for gene expression analysis, with expression of *ERF6* and *LOX2* normalized to the expression of *PP2AA3*. Bacterial growth in WT, (**b**) *ein2* and (**d**) *aos1* mutant plants (n = 4) three days after vacuum-infiltration with *Pst* DC3000. (**e**) ABA metabolite levels in plants (n = 4) 24 h after vacuum-infiltration with mock or *Pst* DC3000. All data are representative of three independent experiments and are presented as the mean ± s.e.m., with n = biological replicates. Letters indicate statistical significance based on a two-factor ANOVA with Tukey's HSD post hoc analysis ($p$-value < 0.05); samples sharing letters are not significantly different. "ns" indicates no statistical significance.

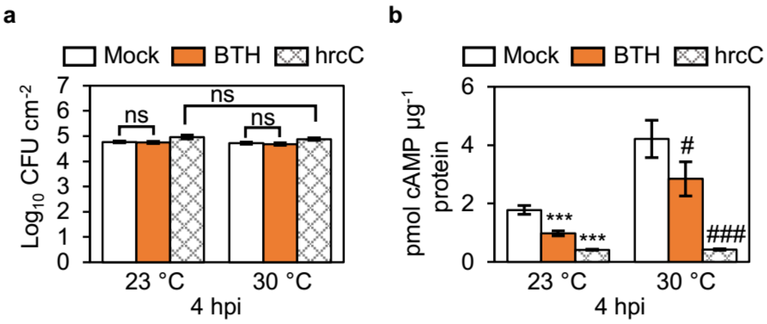


**Supplementary Figure 15. Bacterial populations and effector translocation in plants treated with mock or BTH.** (**a**) Bacterial growth in plants (n = 4) 4 hpi; tissue was collected from the same plants as were used to measure cAMP data presented in Fig. 7g. Plants were pre-treated with mock or BTH 24 h before syringe-infiltration with *Pst* DC3000($P_{nptII}$::*avrPto-CyaA*). Additional mock-treated plants (n = 4 for each temperature) were infiltrated with *hrcC*$^-$ ($P_{nptII}$::*avrPto-CyaA*) as a negative control. (**b**) Translocation of bacterial effector proteins in plants (n = 4) pre-treated with mock or BTH 24 h before syringe-infiltration with *Pst* DC3000($P_{nptII}$::*avrPto-CyaA*) or *hrcC*$^-$($P_{nptII}$::*avrPto-CyaA* strains). Tissue was collected at 4 hpi for quantification of cAMP, which was normalized by total protein. Data are presented as the mean ± s.e.m. with n = biological replicates. Data in (b) and Fig. 7g are two of three independent experiments. In (a), "ns" indicates no statistical significance based on a Student's *t*-test (*p*-value > 0.05). Symbols in (b) denote statistical significance based on a one-factor ANOVA with Dunnett's post hoc analysis (***, ### *p*-value < 0.001, # *p*-value < 0.05) using the mock-treated sample at each temperature as the means for comparison.

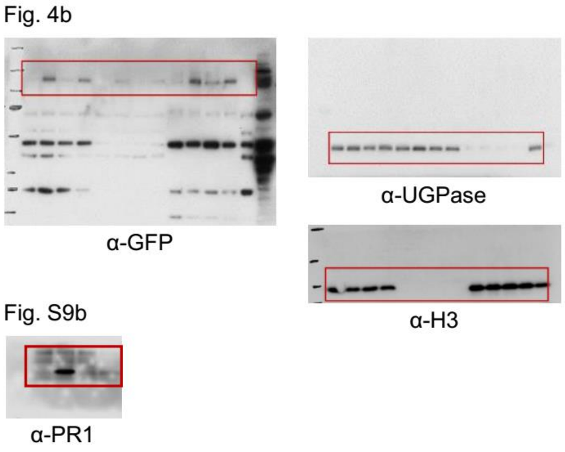


**Supplementary Figure 16. Uncropped western blot images.** Uncropped scans of western blots shown in Fig. 4b and Supplementary Fig. 9b. Red boxes indicate cropped sections used in figures. Images were edited to remove saturation to show in grayscale. Marker lines were drawn on some X-ray scans to show bands in PAGE ruler to determine band size. Last lane in first blot (α-GFP) has the Precision Plus Protein™ WesternC™ Standard (Bio Rad).

## Supplementary Tables

**Supplementary Table 1. Functional annotations for differentially expressed genes based on 23 °C Mock vs. 30 °C Mock analysis.**

| Category | GO term | Up at 30 °C | Down at 30 °C |
|---|---|---|---|
| **Biotic Stress** | Defence | | 30 |
| | Chitin | | 16 |
| | Fungus | | 13 |
| | Bacterium | | 10 |
| | SA | | 6 |
| | ET | | 12 |
| | JA | | 11 |
| | Cell wall | | 10 |
| **Abiotic stress** | Abiotic stress | 17 | 22 |
| | Response to oxygen | 10 | 38 |
| | Temperature/heat | 14 | |

The Gene ontology (GO) term is provided in the second column. Subsequent columns provide the number of genes for the corresponding GO term that are either up- or down-regulated at 30 °C relative to expression levels observed at 23 °C. There is some redundancy of genes across categories (e.g. all temperature genes are also annotated as involved in abiotic stress). GO analysis was conducted using DAVID (https://david.ncifcrf.gov/). For data used to compile this table, see Supplementary Data 2.

**Supplementary Table 2. Distribution of differentially expressed genes in response to BTH by group and cluster.**

**a**

| | Cluster → | 1 | 2 | 3 | 4 | 5 | 6 | 7 | 8 | 9 | Total |
|---|---|---|---|---|---|---|---|---|---|---|---|
| Group | A | 121 | 344 | 318 | 160 | 12 | 1 | 0 | | | 956 |
| Group | B | 12 | 36 | 97 | 221 | 266 | 87 | 6 | | | 725 |
| Group | C | 0 | 0 | 1 | 1 | 40 | 80 | 30 | | | 152 |
| Group | D | | | | | | | | 365 | 445 | 810 |
| Group | E | | | | | | | | 149 | 28 | 177 |
| | Total | 133 | 380 | 416 | 382 | 318 | 168 | 36 | 514 | 473 | |

**b**

| | Cluster → | 1 | 2 | 3 | 4 | 5 | 6 | 7 | 8 | 9 | Total |
|---|---|---|---|---|---|---|---|---|---|---|---|
| Group | A | 91% | 91% | 76% | 42% | 4% | 1% | | | | 52% |
| Group | B | 9% | 9% | 23% | 58% | 84% | 52% | 17% | | | 40% |
| Group | C | | | | | 13% | 48% | 83% | | | 8% |
| Group | D | | | | | | | | 71% | 94% | 82% |
| Group | E | | | | | | | | 29% | 6% | 18% |
| | Total | 7% | 21% | 23% | 21% | 17% | 9% | 2% | 52% | 48% | |

Number (**a**) or percentage (**b**) of DEGs in each group and cluster. Groups A through C are defined as genes induced by BTH at 23 °C (genes in clusters 1 – 7) that show lower expression at 30 °C (Group A), similar expression levels at 30 °C (Group B) or higher expression at 30 °C (Group C). Groups D and E are defined as genes suppressed by BTH at 23 °C (genes in clusters 8 and 9) that show higher expression levels at 30 °C. Percentage totals are calculated based on the total number of genes within the cluster or group divided by the total number of induced (1,833) or suppressed (987) genes. For data used to compile this table, see Supplementary Data 1.

**Supplementary Table 3. Functional annotations for differentially expressed genes based on BTH vs. Mock analysis.**

**a**

| Category | GO term | 1 | 2 | 3 | 4 | 5 | 6 | 7 | 8 | 9 |
|---|---|---|---|---|---|---|---|---|---|---|
| **Biotic Stress** | Defence | | | 40 | 53 | 54 | 21 | | | |
| | Cell death | | | 20 | 20 | 21 | | | | |
| | Bacterium | 7 | 13 | 15 | 17 | 14 | | | | |
| | Chitin | | | | 16 | 32 | 20 | 3 | | |
| | Callose | | | | | | | | | |
| | SA/SAR | | | 10 | 19 | | | | | |
| | ET | | | | | 9 | 17 | | | |
| | JA | | | | | 8 | | | | |
| **Abiotic stress** | Abiotic stress | | | | | | 17 | | | |
| | ABA | | | | 13 | | 7 | | | |
| | heat | 7 | 8 | | | | | | | |
| **Growth** | Chloroplast part | | | | | | | | 34 | 45 |
| | photosynthesis | | | | | | | | | 26 |
| | auxin | | | | | | | | 10 | 12 |

**b**

| Category | GO term | A | B | C | D |
|---|---|---|---|---|---|
| **Biotic Stress** | Defence | 70 | 53 | 77 | |
| | Cell death | 29 | 20 | 24 | |
| | Bacterium | 35 | 17 | 17 | |
| | Chitin | | 16 | 55 | |
| | Callose | 4 | | 5 | |
| | SA/SAR | 17 | 16 | | |
| | ET | | | 28 | |
| | JA | | | 13 | |
| **Abiotic stress** | Abiotic stress | | | 41 | 65 |
| | ABA | | 13 | | |
| | heat | 17 | | | |
| **Growth** | Chloroplast part | | | | 79 |
| | photosynthesis | | | | 30 |
| | auxin | 5 | | | 52 |

The GO term is provided in the second column. Subsequent columns provide the number of genes within each cluster (**a**) or group (**b**) containing the corresponding GO term. GO analysis was conducted using DAVID (https://david.ncifcrf.gov/). For data used to compile this table, see Supplementary Data 3 and 4.

**Supplementary Table 4. Differentially expressed genes involved in SA biosynthesis and signalling.**

| Process | AGI Number | Gene Name | Cluster | Group | References |
|---|---|---|---|---|---|
| **Positive regulation of SA biosynthesis** | AT3G52430 | PAD4 | 3 | A | 1-3 |
| | AT3G48090 | EDS1 | 4 | A | 4, 5 |
| | AT3G20600 | NDR1 | 5 | B | 6 |
| | AT5G13320 | PBS3 | 2 | A | 7, 8 |
| | AT5G26920 | CBP60G | 4 | A | 9 |
| | AT1G73805 | SARD1 | 4 | A | 9 |
| | AT4G18170 | WRKY28 | 5 | B | 10 |
| | AT2G46400 | WRKY46 | 4 | A | 10 |
| | AT1G74710 | ICS1, SID2 | 1 | A | 11, 12 |
| | AT4G39030 | EDS5, SID1 | 1 | A | 11 |
| | AT4G14400 | ACD6 | 4 | B | 13 |
| | AT2G13810 | ALD1 | 2 | A | 14, 15 |
| | AT1G64280 | NPR1, SAI1, NIM1 | 4 | B | 2 |
| **Negative regulation of SA biosynthesis** | AT2G40750 | WRKY54 | 4 | B | 16 |
| | AT3G56400 | WRKY70 | 4 | A | 16 |
| | AT1G29690 | CAD1 | 5 | B | 17 |
| | AT2G39660 | BIK1 | 4 | B | 18 |
| | AT1G28380 | NSL1 | 5 | B | 19 |
| | AT1G64280 | NPR1, SAI1, NIM1 | 4 | B | 2, 20 |
| | AT1G52890 | ANAC019 | 5 | B | 21 |
| | AT3G15500 | ANAC055 | 5 | B | 21 |
| | AT4G27410 | ANAC072 | 5 | B | 21 |
| | AT1G32640 | MYC2 | 7 | C | 21 |
| **Positive role in SA signalling/SAR** | AT1G64280 | NPR1, SAI1, NIM1 | 4 | B | 20, 22, 23 |
| | AT5G55170 | SUMO3 | 4 | B | 24 |
| | AT1G22070 | TGA3 | 3 | A | 25, 26 |
| | AT5G06960 | TGA5, OBF5 | 2 | A | 27 |
| | AT4G31800 | WRKY18 | 4 | A | 16 |
| | AT4G23810 | WRKY53 | 5 | C | 16 |
| | AT2G40750 | WRKY54 | 4 | B | 16 |
| | AT3G56400 | WRKY70 | 4 | A | 16 |
| | AT2G13810 | ALD1 | 2 | A | 14, 28 |
| | AT1G19250 | FMO1 | 3 | A | 29 |
| | AT2G14610 | PR1 | 2 | A | 30 |
| | AT3G57260 | PR2, BGL2 | 4 | A | 30 |
| | AT1G75040 | PR5 | 4 | A | 30 |
| **Negative role in SA signalling/SAR** | AT5G45110 | NPR3 | 4 | B | 31, 32 |
| | AT1G02450 | NIMIN1 | 4 | A | 33, 34 |
| | AT3G25882 | NIMIN-2 | 4 | A | 33, 34 |

| | | | | | |
|---|---|---|---|---|---|
| | AT4G31800 | WRKY18 | 4 | A | 35 |
| | AT5G22570 | WRKY38 | 4 | B | 16, 36 |
| | AT3G01080 | WRKY58 | 4 | A | 16 |
| | AT2G25000 | WRKY60 | 3 | A | 35 |
| | AT5G01900 | WRKY62 | 3 | A | 36, 37 |
| | AT5G04340 | ZAT6 | 6 | B | 38 |

Genes are identified based on their Arabidopsis Genome Initiative (AGI) number and commonly used gene name. Cluster numbers are based on k-means clustering. Group classifications are based on expression level differences (2-fold cut-off) between the BTH-treated samples at 23 °C and 30 °C. See Supplementary Data 1 for spreadsheet containing full list of DEGs.

**Supplementary Table 5. Transcription factors implicated in regulation of genes in BTH vs. Mock analyses.**

**a**

| cluster 2 | cluster 3 | cluster 4 | cluster 5 | cluster 6 | cluster 8 | cluster 9 |
|---|---|---|---|---|---|---|
| TGA5 | | TGA5 | TGA5 | CAMTA2 | PIF1 | PIF1 |
| TGA6 | | TGA6 | TGA6 | CAMTA3 | PIF3 | PIF3 |
| TGA2 | | | | BZR1 | PIF4 | PIF4 |
| ANAC55 | ANAC55 | ANAC55 | ANAC55 | ABF1 | PIF5 | PIF5 |
| | | WRKY48 | | ABF2 | EDT1 | EDT1 |
| | | WRKY33 | WRKY33 | ABF3 | MYC2 | MYC2 |
| | WRKY51 | WRKY51 | WRKY51 | ABI5 | MYC3 | MYC3 |
| | WRKY60 | WRKY60 | WRKY60 | | MYC4 | ATHB6 |
| | WRKY40 | WRKY40 | WRKY40 | | JAM2 | |
| WRKY47 | WRKY47 | WRKY47 | WRKY47 | | BIM2 | |
| WRKY8 | WRKY8 | WRKY8 | WRKY8 | WRKY8 | BIM3 | |
| WRKY12 | WRKY12 | WRKY12 | WRKY12 | WRKY12 | BEE2 | |
| WRKY14 | WRKY14 | WRKY14 | WRKY14 | WRKY14 | | |
| WRKY17 | WRKY17 | WRKY17 | WRKY17 | WRKY17 | | |
| WRKY21 | WRKY21 | WRKY21 | WRKY21 | WRKY21 | | |
| WRKY23 | WRKY23 | WRKY23 | WRKY23 | WRKY23 | | |
| WRKY30 | WRKY30 | WRKY30 | WRKY30 | WRKY30 | | |
| WRKY38 | WRKY38 | WRKY38 | WRKY38 | WRKY38 | | |
| WRKY45 | WRKY45 | WRKY45 | WRKY45 | WRKY45 | | |
| WRKY57 | WRKY57 | WRKY57 | WRKY57 | WRKY57 | | |
| WRKY62 | WRKY62 | WRKY62 | WRKY62 | WRKY62 | | |
| WRKY75 | WRKY75 | WRKY75 | WRKY75 | WRKY75 | | |
| | WRKY2 | WRKY2 | WRKY2 | WRKY2 | | |
| | WRKY11 | WRKY11 | WRKY11 | WRKY11 | | |
| | WRKY15 | WRKY15 | WRKY15 | WRKY15 | | |
| | WRKY18 | WRKY18 | WRKY18 | WRKY18 | | |
| | WRKY25 | WRKY25 | WRKY25 | WRKY25 | | |
| | WRKY43 | WRKY43 | WRKY43 | WRKY43 | | |
| | WRKY63 | WRKY63 | WRKY63 | WRKY63 | | |

**b**

| Group A | Group B | Group C | Group D |
|---|---|---|---|
| TGA5 | CAMTA2 | CAMTA2 | TCP4 |
| TGA6 | CAMTA3 | CAMTA3 | TCP15 |
| NTL9 | NTL9 | GBF1 | TCP16 |
| | CCA1 | GBF3 | TCP23 |
| | RVE1 | GBF4 | GBF4 |
| | RVE6 | ABI5 | ABI5 |

| | | | |
|---|---|---|---|
| | ABF3 | ABF3 | |
| WRKY2 | WRKY2 | ABF1 | ABF1 |
| WRKY8 | WRKY8 | AREB1 | AREB1 |
| WRKY11 | WRKY11 | | MYC3 |
| WRKY12 | WRKY12 | MYC2 | MYC2 |
| WRKY14 | WRKY14 | MYC4 | MYC4 |
| WRKY15 | WRKY15 | JAM1 | JAM1 |
| WRKY17 | WRKY17 | JAM2 | JAM2 |
| WRKY18 | WRKY18 | | PIF1 |
| WRKY21 | WRKY21 | PIF3 | PIF3 |
| WRKY23 | WRKY23 | PIF4 | PIF4 |
| WRKY25 | WRKY25 | | PIF5 |
| WRKY30 | WRKY30 | BZR1 | BZR1 |
| WRKY38 | WRKY38 | HY5 | BIM2 |
| WRKY40 | WRKY40 | EDT1 | BIM3 |
| WRKY43 | WRKY43 | BEE2 | CIB5 |
| WRKY45 | WRKY45 | BES1 | HBI1 |
| WRKY47 | WRKY47 | SPT | SPT |
| WRKY51 | WRKY51 | | UNE10 |
| WRKY60 | WRKY60 | | SPL8 |
| WRKY57 | WRKY57 | WRKY57 | AGL8 |
| WRKY62 | WRKY62 | WRKY62 | SEP4 |
| WRKY75 | WRKY75 | WRKY75 | SEP3 |
| WRKY63 | WRKY63 | | BPE |
| | WRKY1 | | |
| | WRKY27 | | |
| | WRKY29 | | |
| | WRKY33 | | |
| | WRKY48 | | |

Analysis of Motif Enrichment (AME; http://meme-suite.org/tools/ame) was conducted by cluster (**a**) and by group (**b**) as described in Supplementary Note 1. For each analysis, a subset of TFs, as implicated by enrichment of the motif to which they bind, was compiled based on known relevance to SA, ABA, JA, and growth-related processes. No enriched motifs were identified for clusters 1 and 7. See Supplementary Data 5 for html files with actual data output for each analysis.

**Supplementary Table 6. Primer sequences.**

| **AGI Number (Gene name)** | **Primer name** | **Primer sequence (5'-3')** | **Purpose** |
|---|---|---|---|
| NA | SAIL_LB3 | TAGCATCTGAATTTCATAACCAATCTCGATACAC | Genotyping |
| AT1G64280 (*NPR1*) | SAIL708F09_LP | ATTTGTTTGAAGCACACCTGC | Genotyping |
| | SAIL708F09_RP | CTCTCAAAGGCCGACTATGTG | Genotyping |
| NA | SALK_LBb1.3 | ATTTTGCCGATTTCGGAAC | Genotyping |
| AT1G32640 (*MYC2*) | MYC2_GT_LP | GCTACAACCAACGATGAATC | Genotyping |
| | MYC2_GT_RP | TCATCAACAGCGTCATCCGA | Genotyping |
| NA | GABI Kat_LB1 | ATAACGCTGCGGACATCTACATT | Genotyping |
| AT5G46760 (*MYC3*) | MYC3_GT_LP | GTTAGATCAGCTGCGAATGATTCGG | Genotyping |
| | MYC3_GT_RP | CTCCGACTTTCGTCATCAAAGCAAC | Genotyping |
| AT4G17880 (*MYC4*) | MYC4_GT_LP | GGATCCATGTCTCCGACGAATGTTCAAGTA | Genotyping |
| | MYC4_GT_RP | TCTCTCACAACTTGATCCAGCTAA | Genotyping |
| AT1G64280 (*NPR1*) | NPR1_F | agaattcATGGACACCACCATTGATGGA | Cloning |
| | NPR1_R | agtcgacCCGACGACGATGAGAGARTTTAC | Cloning |
| *NPR1* promoter | pNPR1_F | cgcggccgcTCGTTTGTTTTCCGTTTTGTTCTGA | Cloning |
| | pNPR1_R | agaattcCAACAGGTTCCGATGAATTGAAAT | Cloning |
| AT1G64280 (*NPR1*) | NPR1-NT F | AGGATCCATGGACACCACCATTGATGG | RT-PCR |
| | NPR1-CT R | AGCGGCCGCTCACCGACGACGATGAGAGA | RT-PCR |
| At2g14610 (*PR1*) | PR1-RT-F | GTTCACAACCAGGCACGA | RT-PCR |
| | PR1-RT-R | CACCTCACTTTGGCACATCC | RT-PCR |
| AT4G05320 (*UBQ10*) | UBQ10-RT-F | ACCCTCCACTTGGTCCTCA | RT-PCR |
| | UBQ10-RT-R | AGTTTTCCCAGTCAACGTCTT | RT-PCR |
| At1G13320 (*PP2AA3*) | PP2AA3_qRT_F1 | GGTTACAAGACAAGGTTCACTC | qPCR |
| | PP2AA3_qRT_R1 | CATTCAGGACCAAACTCTTCAG | qPCR |
| At2g14610 (*PR1*) | PR1_qRT_F1 | GGCTAACTACAACTACGCTG | qPCR |
| | PR1_qRT_R1 | TCTCGTTCACATAATTCCCAC | qPCR |
| At1g74710 (*ICS1*) | ICS1_qRT_F2 | ACTTACTAACCAGTCCGAAAGACGA | qPCR |
| | ICS1_qRT_R2 | ACAACAACTCTGTCACATATACCGT | qPCR |
| AT5G03280 (*EIN2*) | EIN2_qRT_F1 | TGGGGAAAGTACACTTACGTTCTCAA | qPCR |
| | EIN2_qRT_R1 | ATTCCGTTAGCTGAAGTCGGACT | qPCR |
| AT4G17490 (*ERF6*) | ERF6_qRT_F1 | AGAGGAGAAGAGGCATTACAGAG | qPCR |
| | ERF6_qRT_R1 | GAGCCAAACACGAGTTCCAC | qPCR |
| AT4G17880 (*MYC2*) | MYC2_qRT_F1 | GAAACTCCAAATCAAGAACCAG | qPCR |
| | MYC2_qRT_R1 | ATCTTCACTTCAATCTCCATCC | qPCR |
| At3g45140 (*LOX2*) | LOX2_qRT_F | GCCGTTAATAAGCAAACTAGAC | qPCR |
| | LOX2_qRT_R | TTCTTTAGAGCCTCATCAACTG | qPCR |
| AT3G14440 (*NCED3*) | NCED3_qRT_F1 | AAAGCCATCGGTGAGCTTCA | qPCR |
| | NCED3_qRT_R1 | GCAGCTCTGGCGTAGAATAGC | qPCR |

## Supplementary Notes

### Supplementary Note 1

**RNA sequencing and data analysis.** Extracted RNA was checked for purity using a BioAnalyzer Agilent 2100 and three biological replicates of each treatment type were selected based on having a RNA Integrity Number (RIN) score around 7. The final twelve samples were submitted to the Research Technology Support Facility at Michigan State University for preparation of next-generation sequencing libraries. Pooled samples were loaded on two lanes of an Illumina HiSeq 2500 Rapid Run flow cell (v1) and sequenced in a 1 x 50 bp single end format using Rapid SBS reagents. Base calling was done by Illumina Real Time Analysis (RTA) v1.18.61 and output of RTA was de-multiplexed and converted to FastQ format using Illumina Bcl2fastq v1.8.4. Due to inadvertent exclusion of sample 6, a second pool was generated and run on one lane of a Rapid Run flow cell, and sample 6 was also run individually on a MiSeq flow cell.

RNA-seq reads were cleaned and trimmed using Trimmomatic[39] and were aligned to the *Arabidopsis thaliana* genome assembly (TAIR10) using the STAR alignment program, allowing only unique alignments[40]. Read counts were obtained for each gene using the featureCounts function from the Rsubread package in R[41], and subsequent count data were normalized using TMM using the limma package in R[42]. Genes with average counts less than 10 across all samples were discarded. The count data were normalized with normalization factors calculated by the function calcNormFactors in the package edgeR in R, and log-transformed by the function voom in the package limma in R to yield $\log_2$ counts per million[42]. The expression data were fit to a linear model (treatment:temp + replicate) by using the function lmFit in the limma package in R. The eBayes function in the limma package in R was used for variance shrinkage in calculation of the *p*-values, which were then used to calculate the Storey's *q*-values using the qvalue function in the qvalue package in R. Differentially expressed genes were identified with a

*q*-value < 0.01. Results were then filtered for those genes that exhibited a $\log_2$-fold change greater than 2. Gene ontology analysis was done for genes in each cluster using The Database for Annotation, Visualization and Integrated Discovery (DAVID)[43]. For motif enrichment analysis, the 1,000 bp upstream of the transcription start sites of the selected genes were tested for enrichment of the known *cis* elements using AME[44]. The heatmap was generated with CLUSTER using k-Means (k=9), and visualized by TREEVIEW[45].

**Supplementary Note 2**

**Confirmation of a T-DNA knock-out allele for NPR1.** We identified a *npr1* mutant allele, SAIL_708F09, containing a T-DNA insertion in the third exon of the *NPR1* gene (Supplementary Fig. 7a). RT-PCR was used to confirm this allele has a complete loss of *NPR1* transcript and loss of *PR1* gene induction by BTH (Supplementary Fig. 7d). Bacterial growth in mock- and BTH-treated plants was assessed to confirm enhanced susceptibility relative to WT and loss of BTH-mediated protection (Supplementary Fig. 7c). We named this allele *npr1-6*.

**Preparation, selection and complementation analysis of transgenic lines.** The full length coding sequence of *NPR1* without the stop codon was PCR-amplified using NPR1_F and NPR1_R primers (Supplementary Table 6) and cloned into *EcoR*I/*Xho*I sites of pENJAZ9C[46] to create pEN*NPR1C*, a Gateway compatible entry vector. Next, a 2.3 kb DNA fragment containing the *NPR1* promoter was PCR-amplified using pNPR1_F and pNPR1_R primers (Supplementary Table 6) and cloned into *Not*I/*EcoR*I sites of pEN*NPR1C* to create pEN*pNPR1C::NPR1*. Then, the *pNPR1::NPR1* construct was transferred by LR recombination into the binary expression vector pGWB540 to create the *pNPR1::NPR1-YFP* construct (Supplementary Fig. 7b). The correct construct was confirmed by sequencing and introduced into *Agrobacterium tumefaciens* (GV3101) by electroporation. GV3101 clones containing the gene fusion construct were selected on LB medium containing rifampicin (Rif, 100 mg $L^{-1}$), spectinomycin (50 mg $L^{-1}$), and gentamycin (25 mg $L^{-1}$) antibiotics and used to transform *npr1-6* by floral dipping[47]. T1 seeds were plated on ½X Murashige and Skoog, 5 mM MES, 0.7 % Bacto agar plates (1/2 MMS) containing hygromycin and resistant seedlings were transplanted to soil. Ten T1 plants were selected for protein extraction, and transgene expression was assessed using Western blot analysis using an α-GFP primary antibody (1:5,000, Abcam, data not shown). T2 seeds were collected from the ten T1 lines and ~100 seeds each were sown on ½ MMS plus 1 % sucrose (1/2 MMSS) plates containing hygromycin (25 mg $L^{-1}$) to ascertain segregation ratios. Resistant T2 seedlings from lines exhibiting a 3:1 segregation ratio were then screened for induction by

BTH using confocal microscopy (data not shown). Lines showing strong induction were transplanted to soil. Homozygous T3 lines were selected by screening for 100 % resistance to hygromycin. BTH protection assays were conducted in three independent lines to confirm complementation of the *npr1-6* knockout mutation (Supplementary Fig. 7c). RT-PCR was also used to confirm recovery of *NPR1* expression and BTH-induction of *PR1* in the NPR1-Y1 line used for experiments (Supplementary Fig. 7d).

For generation of *p35S::YFP* transgenic plants, the GATEWAY cassette in pEARLEYGATE104[48] was removed by XmaI digestion, and the resulting linearized vector was re-ligated to create *pJYP35S:YFP*. This construct was used for Agrobacterium-mediated transformation of Arabidopsis Col-0 wild-type plants by floral dipping[47]. A homozygous T3 line with the single T-DNA insertion was selected for the further experiments.

**Supplementary Note 3**

***Pst* DC3000 Inoculum Preparation.** *Pst* DC3000 was streaked from a frozen glycerol stock onto a LM (10.0 g Bacto Tryptone, 6.0 g Bacto yeast extract, 1.5 g $K_2HPO_4$, 0.6 g NaCl, 0.4 g $MgSO_4$ * 7 $H_2O$ $L^{-1}$) + Rif (100 mg $L^{-1}$) plate and grown in the dark for two days at room temperature until single colonies were formed. Colonies from this plate were streaked onto a fresh LM+Rif plate and grown in the dark for one day at room temperature, after which 100 µl sterile LM media was added to the plate and the cells were spread evenly and kept in the dark at room temperature overnight to form a lawn. Cells were scraped from this lawn plate and re-suspended in 0.25 mM $MgCl_2$ by incubating at room temperature for 5 min and then vortexing vigorously. A DU800 Spectrophotometer (Beckman Coulter, Inc, Fullerton, California) was used to measure the optical density (OD) of the culture at an absorbance wavelength of 600 nm ($OD_{600}$), and an inoculation culture was prepared by first adjusting the starting culture to $1 \times 10^8$ CFU $ml^{-1}$ ($OD_{600}$ of approximately 0.1) and then preparing 1:10 dilutions to reach the desired inoculum concentration of ~1 – 3 x $10^6$ CFU $ml^{-1}$ ($OD_{600}$ of approximately 0.001). For vacuum-infiltration, Silwet (0.005%) was added to the culture to enhance wetting of the leaves. Serial dilutions of inoculum were plated to determine the actual CFU $ml^{-1}$ of culture used in each experiment.

**Disease and BTH protection assays.** Following temperature acclimation and chemical treatments, syringe- or vacuum-infiltration was used to inoculate plants as previously described[49]. Following infiltration, plants were immediately returned to the test chambers where the leaves were allowed to dry until leaves returned to the pre-infiltration appearance before covering with transparent domes to maintain high humidity. Bacterial quantification was done by harvesting and grinding leaf discs in 0.25 mM $MgCl_2$ and preparing serial dilutions, which were then plated on modified Luria-Bertani liquid medium (LM, 10.0 g Bacto tryptone, 6.0 g Bacto yeast extract, 1.5 g $K_2HPO_4$, 0.6 g NaCl, 0.4 g $MgSO_4 \cdot 7 H_2O$ $L^{-1}$ $H_2O$) + Rifampicin (Rif, 100

mg $ml^{-1}$) plates and kept for 24 h at 30 °C. CFUs were counted and the CFU $cm^{-2}$ calculated as (CFUs * total dilution)/(vol plated)/leaf area harvested.

## Supplementary References


1. Jirage, D. *et al. Arabidopsis thaliana PAD4* encodes a lipase-like gene that is important for salicylic acid signaling. *Proc. Natl. Acad. Sci. U. S. A.* **96**, 13583-13588 (1999).
2. Lu, H. *et al.* Genetic analysis of *acd6-1* reveals complex defense networks and leads to identification of novel defense genes in Arabidopsis. *Plant J.* **58**, 401-412 (2009).
3. Zhou, N., Tootle, T.L., Tsui, F., Klessig, D.F. & Glazebrook, J. *PAD4* functions upstream from salicylic acid to control defense responses in Arabidopsis. *Plant Cell* **10**, 1021-1030 (1998).
4. Falk, A. *et al.* EDS1, an essential component of *R* gene-mediated disease resistance in Arabidopsis has homology to eukaryotic lipases. *Proc. Natl. Acad. Sci. U. S. A.* **96**, 3292-3297 (1999).
5. Feys, B.J., Moisan, L.J., Newman, M.-A. & Parker, J.E. Direct interaction between the *Arabidopsis* disease resistance signaling proteins, EDS1 and PAD4. *The EMBO Journal* **20**, 5400-5411 (2001).
6. Shapiro, A.D. & Zhang, C. The role of NDR1 in avirulence gene-directed signaling and control of programmed cell death in Arabidopsis. *Plant Physiol.* **127**, 1089-1101 (2001).
7. Jagadeeswaran, G. *et al.* Arabidopsis *GH3-LIKE DEFENSE GENE 1* is required for accumulation of salicylic acid, activation of defense responses and resistance to *Pseudomonas syringae*. *Plant J.* **51**, 234-246 (2007).
8. Nobuta, K. *et al.* The GH3 acyl adenylase family member PBS3 regulates salicylic acid-dependent defense responses in Arabidopsis. *Plant Physiol.* **144**, 1144-1156 (2007).
9. Zhang, Y.X. *et al.* Control of salicylic acid synthesis and systemic acquired resistance by two members of a plant-specific family of transcription factors. *Proc. Natl. Acad. Sci. U. S. A.* **107**, 18220-18225 (2010).
10. van Verk, M.C., Bol, J.F. & Linthorst, H.J.M. WRKY transcription factors involved in activation of SA biosynthesis genes. *BMC Plant Biol.* **11**, doi: 10.1186/1471-2229-1111-1189 (2011).
11. Nawrath, C. & Metraux, J.P. Salicylic acid induction-deficient mutants of Arabidopsis express *PR-2* and *PR-5* and accumulate high levels of camalexin after pathogen inoculation. *Plant Cell* **11**, 1393-1404 (1999).
12. Wildermuth, M.C., Dewdney, J., Wu, G. & Ausubel, F.M. Isochorismate synthase is required to synthesize salicylic acid for plant defence. *Nature* **414**, 562-565 (2001).
13. Lu, H., Rate, D.N., Song, J.T. & Greenberg, J.T. ACD6, a novel ankyrin protein, is a regulator and an effector of salicylic acid signaling in the Arabidopsis defense response. *Plant Cell* **15**, 2408-2420 (2003).
14. Cecchini, N.M., Jung, H.W., Engle, N.L., Tschaplinski, T.J. & Greenberg, J.T. ALD1 regulates basal immune components and early inducible defense responses in Arabidopsis. *Mol. Plant Microbe Interact.* **28**, 455-466 (2015).
15. Ng, G.N. *et al.* Genetic dissection of salicylic acid-mediated defense signaling networks in *Arabidopsis*. *Genetics* **189**, 851-859 (2011).
16. Wang, D., Amornsiripanitch, N. & Dong, X.N. A genomic approach to identify regulatory nodes in the transcriptional network of systemic acquired resistance in plants. *PLoS Path.* **2**, 1042-1050 (2006).
17. Morita-Yamamuro, C. *et al.* The *Arabidopsis* gene *CAD1* controls programmed cell death in the plant immune system and encodes a protein containing a MACPF domain. *Plant Cell Physiol.* **46**, 902-912 (2005).
18. Veronese, P. *et al.* The membrane-anchored *BOTRYTIS-INDUCED KINASE1* plays distinct roles in *Arabidopsis* resistance to necrotrophic and biotrophic pathogens. *Plant Cell* **18**, 257-273 (2006).

19. Noutoshi, Y. *et al.* Loss of *NECROTIC SPOTTED LESIONS 1* associates with cell death and defense responses in *Arabidopsis thaliana*. *Plant Mol. Biol.* **62**, 29-42 (2006).
20. Delaney, T.P., Friedrich, L. & Ryals, J.A. *Arabidopsis* signal-transduction mutant defective in chemically and biologically induced disease resistance. *Proc. Natl. Acad. Sci. U. S. A.* **92**, 6602-6606 (1995).
21. Zheng, X.Y. *et al.* Coronatine promotes *Pseudomonas syringae* virulence in plants by activating a signaling cascade that inhibits salicylic acid accumulation. *Cell Host Microbe* **11**, 587-596 (2012).
22. Cao, H., Bowling, S.A., Gordon, A.S. & Dong, X.N. Characterization of an Arabidopsis mutant that is nonresponsive to inducers of systemic acquired-resistance. *Plant Cell* **6**, 1583-1592 (1994).
23. Shah, J., Tsui, F. & Klessig, D.F. Characterization of a salicylic acid-insensitive mutant (*sai1*) of *Arabidopsis thaliana*, identified in a selective screen utilizing the SA-inducible expression of the *tms2* gene. *Mol. Plant Microbe Interact.* **10**, 69-78 (1997).
24. Saleh, A. *et al.* Posttranslational modifications of the master transcriptional regulator NPR1 enable dynamic but tight control of plant immune responses. *Cell Host Microbe* **18**, 169-182 (2015).
25. Johnson, C., Boden, E. & Arias, J. Salicylic acid and NPR1 induce the recruitment of trans-activating TGA factors to a defense gene promoter in Arabidopsis. *Plant Cell* **15**, 1846-1858 (2003).
26. Zhou, J.M. *et al.* NPR1 differentially interacts with members of the TGA/OBF family of transcription factors that bind an element of the *PR-1* gene required for induction by salicylic acid. *Mol. Plant Microbe Interact.* **13**, 191-202 (2000).
27. Zhang, Y., Tessaro, M.J., Lassner, M. & Li, X. Knockout analysis of Arabidopsis transcription factors *TGA2*, *TGA5*, and *TGA6* reveals their redundant and essential roles in systemic acquired resistance. *Plant Cell* **15**, 2647-2653 (2003).
28. Song, J.T., Lu, H., McDowell, J.M. & Greenberg, J.T. A key role for *ALD1* in activation of local and systemic defenses in *Arabidopsis*. *Plant J.* **40**, 200-212 (2004).
29. Mishina, T.E. & Zeier, J. The Arabidopsis flavin-dependent monooxygenase FMO1 is an essential component of biologically induced systemic acquired resistance. *Plant Physiol.* **141**, 1666-1675 (2006).
30. Uknes, S. *et al.* Acquired resistance in Arabidopsis. *Plant Cell* **4**, 645-656 (1992).
31. Fu, Z.Q. *et al.* NPR3 and NPR4 are receptors for the immune signal salicylic acid in plants. *Nature* **486**, 228-232 (2012).
32. Zhang, Y. *et al.* Negative regulation of defense responses in Arabidopsis by two *NPR1* paralogs. *Plant J.* **48**, 647-656 (2006).
33. Hermann, M. *et al.* The Arabidopsis NIMIN proteins affect NPR1 differentially. *Front. Plant Sci.* **4**, doi: 10.3389/fpls.2013.00088 (2013).
34. Weigel, R.R., Pfitzner, U.M. & Gatz, C. Interaction of NIMIN1 with NPR1 modulates *PR* gene expression in Arabidopsis. *Plant Cell* **17**, 1279-1291 (2005).
35. Xu, X., Chen, C., Fan, B. & Chen, Z. Physical and functional interactions between pathogen-induced Arabidopsis WRKY18, WRKY40, and WRKY60 transcription factors. *Plant Cell* **18**, 1310-1326 (2006).
36. Kim, K.-C., Lai, Z., Fan, B. & Chen, Z. Arabidopsis WRKY38 and WRKY62 Transcription Factors Interact with Histone Deacetylase 19 in Basal Defense. *Plant Cell* **20**, 2357-2371 (2008).
37. Mao, P., Duan, M., Wei, C. & Li, Y. WRKY62 transcription factor acts downstream of cytosolic NPR1 and negatively regulates jasmonate-responsive gene expression. *Plant Cell Physiol.* **48**, 833-842 (2007).
38. Shi, H. *et al.* The cysteine2/histidine2-type transcription factor *ZINC FINGER OF ARABIDOPSIS THALIANA6* modulates biotic and abiotic stress responses by activating

salicylic acid-related genes and *C-REPEAT-BINDING FACTOR* Genes in Arabidopsis. *Plant Physiol.* **165**, 1367-1379 (2014).

39. Bolger, A.M., Lohse, M. & Usadel, B. Trimmomatic: a flexible trimmer for Illumina sequence data. *Bioinformatics* **30**, 2114-2120 (2014).
40. Dobin, A. *et al.* STAR: ultrafast universal RNA-seq aligner. *Bioinformatics* **29**, 15-21 (2013).
41. Liao, Y., Smyth, G.K. & Shi, W. The Subread aligner: fast, accurate and scalable read mapping by seed-and-vote. *Nucleic Acids Res.* **41**, doi: 10.1093/nar/gkt1214 (2013).
42. Law, C.W., Chen, Y., Shi, W. & Smyth, G.K. voom: Precision weights unlock linear model analysis tools for RNA-seq read counts. *Genome Biol.* **15**, doi: 10.1186/gb-2014-1115-1182-r1129 (2014).
43. Huang da, W., Sherman, B.T. & Lempicki, R.A. Systematic and integrative analysis of large gene lists using DAVID bioinformatics resources. *Nat. Protoc.* **4**, 44-57 (2009).
44. McLeay, R.C. & Bailey, T.L. Motif enrichment analysis: a unified framework and an evaluation on ChIP data. *BMC Bioinformatics* **11**, doi: 10.1186/1471-2105-1111-1165 (2010).
45. Eisen, M.B., Spellman, P.T., Brown, P.O. & Botstein, D. Cluster Analysis and Display of Genome-Wide Expression Patterns. *Proc. Natl. Acad. Sci. U. S. A.* **95**, 14863-14868 (1998).
46. Yang, D.L. *et al.* Plant hormone jasmonate prioritizes defense over growth by interfering with gibberellin signaling cascade. *Proc. Natl. Acad. Sci. U. S. A.* **109**, E1192-E1200 (2012).
47. Bent, A. *Arabidopsis thaliana* floral dip transformation method. *Methods Mol. Biol.* **343**, 87-103 (2006).
48. Earley, K.W. *et al.* Gateway-compatible vectors for plant functional genomics and proteomics. *Plant J.* **45**, 616-629 (2006).
49. Katagiri, F., Thilmony, R. & He, S.Y. The *Arabidopsis thaliana-Pseudomonas syringae* Interaction. *The Arabidopsis Book*, doi: 10.1199/tab.0039 (2002).
