# Supplementary material for: Dual impact of elevated temperature on plant defence and bacterial virulence in Arabidopsis
Source: Nat Commun. 2017 Nov 27;8:1808. doi: 10.1038/s41467-017-01674-2 (PMC5704021; doi:10.1038/s41467-017-01674-2)
Supplement: Supplementary file 3 — Description of Additional Supplementary Files [file 41467_2017_1674_MOESM3_ESM.pdf]

Description of Supplementary Files

File Name: Supplementary Data 1
Description: Differentially expressed genes from RNA sequencing analysis.

File Name: Supplementary Data 2
Description: Gene ontology analysis of DEGs affected by temperature.

File Name: Supplementary Data 3
Description: Gene ontology analyses of DEGs by cluster.

File Name: Supplementary Data 4
Description: Gene ontology analyses of DEGs by group.

File Name: Supplementary Data 5
Description: Promoter analyses of DEGs by cluster and group.

File Name: Supplementary Data 6
Description: Raw genevestigator output corresponding to Supplementary Figures 11-13.
