# Supplementary material for: Dual impact of elevated temperature on plant defence and bacterial virulence in Arabidopsis
Source: Nat Commun. 2017 Nov 27;8:1808. doi: 10.1038/s41467-017-01674-2 (PMC5704021; doi:10.1038/s41467-017-01674-2)
Supplement: Supplementary file 9 — Supplementary Data 6 [file 41467_2017_1674_MOESM9_ESM.zip › Genevestigator_RawOutput/Cluster2_data.pdf]

Dataset: 19 perturbations (sample selection: SYH)
335 probes (gene selection: SYH_C6)

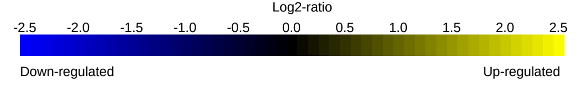

14 of 14 perturbations fulfilled the filter criteria

Filter values for ● AT1G01010 (261585_at)

Arabidopsis thaliana (14)

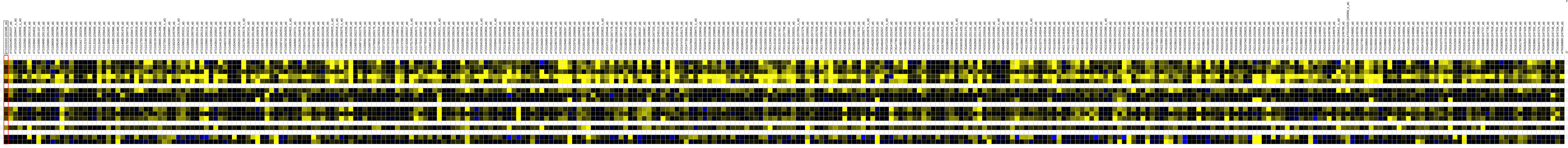

Perturbation	Log2-ratio (no filter)	Fold-Change (no filter)	p-value
▼ Biotic			
A. brassicicola study 3 (Col-0) / mock treated leaf samples (Col-0)	1.16	2.17	0.044
B. cinerea / non-infected rosette leaf samples	0.30	1.25	0.293
H. arabidopsidis study 4 (Col-0) / untreated seedling samples (Col-0)	0.49	1.38	0.219
P. syringae pv. maculicola (Col-0) / mock treated leaf samples (Col-0)	1.95	3.87	<0.001
P. syringae pv. tomato study 3 (DC3000) / mock inoculated leaf samples (24h)	0.51	1.42	<0.001
▼ Chemical			
benzothiadiazole study 3 (Col-0) / untreated (Col-0) plant samples	0.67	1.60	0.001
chitin / mock treated seedlings	0.14	1.09	0.301
H2O2 study 3 (Col-0) / untreated seedlings (Col-0)	0.14	1.10	0.131
▼ Elicitor			
EF-Tu (elf18) study 3 (Col-0) / mock treated seedling samples (Col-0)	0.73	1.65	0.002
FLG22 (1h) / H2O treated leaf samples (1h)	0.37	1.27	0.166
Pep2 (Col-0) / mock treated seedling samples (Col-0)	0.77	1.70	0.003
▼ Hormone			
salicylic acid / mock treated seedlings	1.45	2.76	0.020
▼ Stress			
heat study 4 / untreated plant samples	0.20	1.15	0.010
heat study 11 (Col) / untreated seedling samples (Col)	-0.04	-1.03	0.872

created with GENEVESTIGATOR
