# Supplementary material for: Dual impact of elevated temperature on plant defence and bacterial virulence in Arabidopsis
Source: Nat Commun. 2017 Nov 27;8:1808. doi: 10.1038/s41467-017-01674-2 (PMC5704021; doi:10.1038/s41467-017-01674-2)
Supplement: Supplementary file 9 — Supplementary Data 6 [file 41467_2017_1674_MOESM9_ESM.zip › Genevestigator_RawOutput/Cluster3_2_data.pdf]

Dataset: 19 perturbations (sample selection: SYH)

14 probes (gene selection: SYH_C0_2)

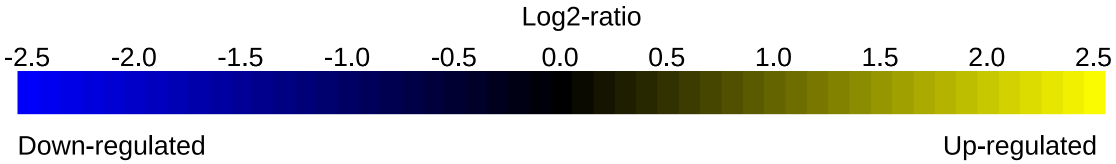

14 of 14 perturbations fulfilled the filter criteria

Filter values for AT5G58620 (247795_at)

no filter no filter

Arabidopsis thaliana (14)

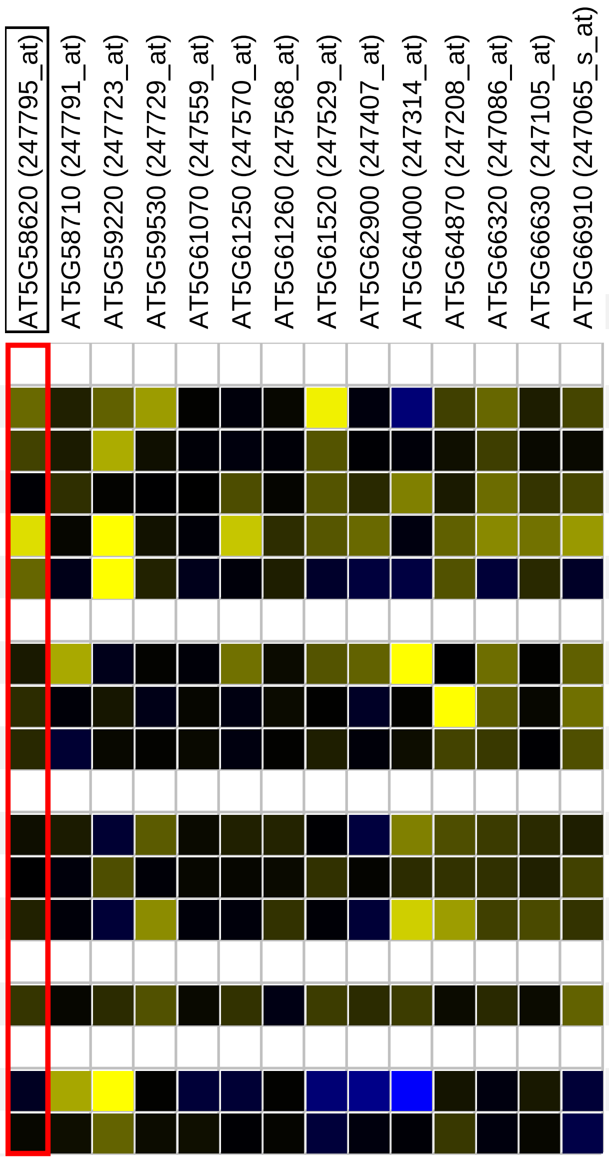

	Log2-ratio	Fold-Change	p-value
▼ Biotic			
A. brassicicola study 3 (Col-0) / mock treated leaf samples (Col-0)	1.05	2.04	0.023
B. cinerea / non-infected rosette leaf samples	0.67	1.80	0.275
H. arabidopsidis study 4 (Col-0) / untreated seedling samples (Col-0)	-0.05	-1.04	0.298
P. syringae pv. maculicola (Col-0) / mock treated leaf samples (Col-0)	2.23	4.68	<0.001
P. syringae pv. tomato study 3 (DC3000) / mock inoculated leaf samples (24h)	1.03	2.03	<0.001
▼ Chemical			
benzothiadiazole study 3 (Col-0) / untreated (Col-0) plant samples	0.26	1.20	0.177
chitin / mock treated seedlings	0.45	1.37	0.083
H2O2 study 3 (Col-0) / untreated seedlings (Col-0)	0.41	1.33	0.068
▼ Elicitor			
EF-Tu (elf18) study 3 (Col-0) / mock treated seedling samples (Col-0)	0.14	1.11	0.321
FLG22 (1h) / H2O treated leaf samples (1h)	-0.02	-1.01	0.933
Pep2 (Col-0) / mock treated seedling samples (Col-0)	0.33	1.26	0.038
▼ Hormone			
salicylic acid / mock treated seedlings	0.54	1.45	0.114
▼ Stress			
heat study 4 / untreated plant samples	-0.35	-1.27	0.024
heat study 11 (Col) / untreated seedling samples (Col)	0.08	1.05	0.641

created with GENEVESTIGATOR
