# Supplementary material for: Dual impact of elevated temperature on plant defence and bacterial virulence in Arabidopsis
Source: Nat Commun. 2017 Nov 27;8:1808. doi: 10.1038/s41467-017-01674-2 (PMC5704021; doi:10.1038/s41467-017-01674-2)
Supplement: Supplementary file 9 — Supplementary Data 6 [file 41467_2017_1674_MOESM9_ESM.zip › Genevestigator_RawOutput/Cluster6_data.pdf]

Dataset: 19 perturbations (sample selection: SYH)

137 probes (gene selection: SYH_C5)

Log2-ratio

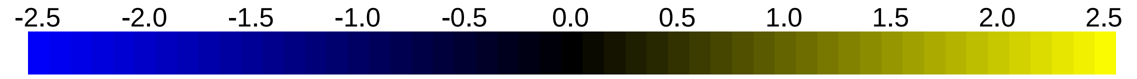

14 of 14 perturbations fulfilled the filter criteria

Filter values for AT1G02360 (259443_at)

Arabidopsis thaliana (14)

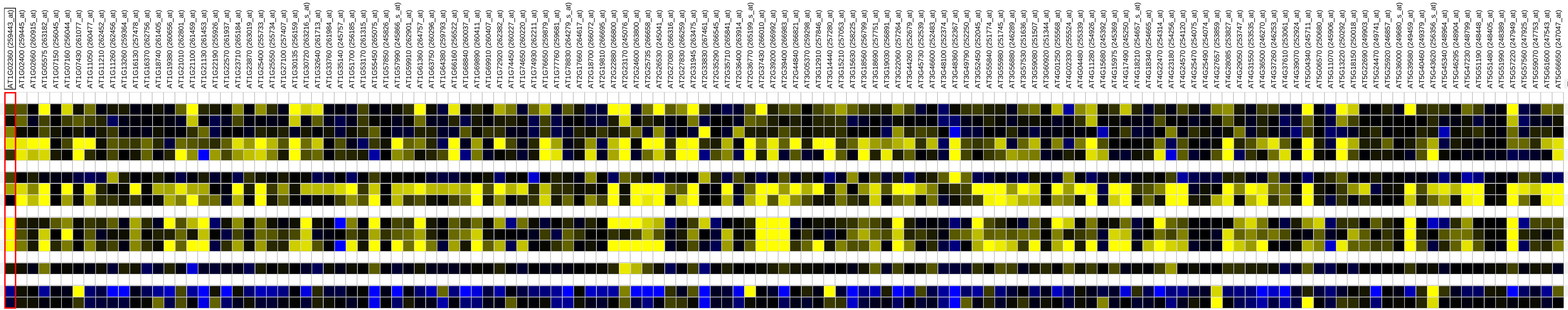

	no filter (Log2-ratio)	Fold-Change	no filter (p-value)
**▼ Biotic**			
A. brassicicola study 3 (Col-0) / mock treated leaf samples (Col-0)	0.70	1.63	0.020
B. cinerea / non-infected rosette leaf samples	0.16	1.15	0.765
H. arabidopsidis study 4 (Col-0) / untreated seedling samples (Col-0)	1.28	2.43	0.006
P. syringae pv. maculicola (Col-0) / mock treated leaf samples (Col-0)	2.30	4.82	0.022
P. syringae pv. tomato study 3 (DC3000) / mock inoculated leaf samples (24h)	0.51	1.46	0.469
**▼ Chemical**			
benzothiadiazole study 3 (Col-0) / untreated (Col-0) plant samples	0.66	1.50	0.254
chitin / mock treated seedlings	1.63	3.12	<0.001
H2O2 study 3 (Col-0) / untreated seedlings (Col-0)	-0.13	-1.10	0.318
**▼ Elicitor**			
EF-Tu (elf18) study 3 (Col-0) / mock treated seedling samples (Col-0)	2.62	6.21	<0.001
FLG22 (1h) / H2O treated leaf samples (1h)	3.56	12.71	0.002
Pep2 (Col-0) / mock treated seedling samples (Col-0)	2.49	5.62	<0.001
**▼ Hormone**			
salicylic acid / mock treated seedlings	0.28	1.22	0.148
**▼ Stress**			
heat study 4 / untreated plant samples	-1.39	-2.63	0.005
heat study 11 (Col) / untreated seedling samples (Col)	-0.56	-1.52	0.084

created with GENEVESTIGATOR
