# Supplementary material for: Dual impact of elevated temperature on plant defence and bacterial virulence in Arabidopsis
Source: Nat Commun. 2017 Nov 27;8:1808. doi: 10.1038/s41467-017-01674-2 (PMC5704021; doi:10.1038/s41467-017-01674-2)
Supplement: Supplementary file 9 — Supplementary Data 6 [file 41467_2017_1674_MOESM9_ESM.zip › Genevestigator_RawOutput/Cluster7_data.pdf]

Dataset: 19 perturbations (sample selection: SYH)

30 probes (gene selection: SYH_C4)

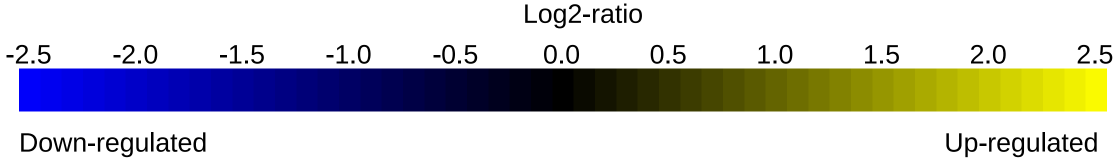

14 of 14 perturbations fulfilled the filter criteria

Filter values for AT1G04360 (263650_at)

Arabidopsis thaliana (14)

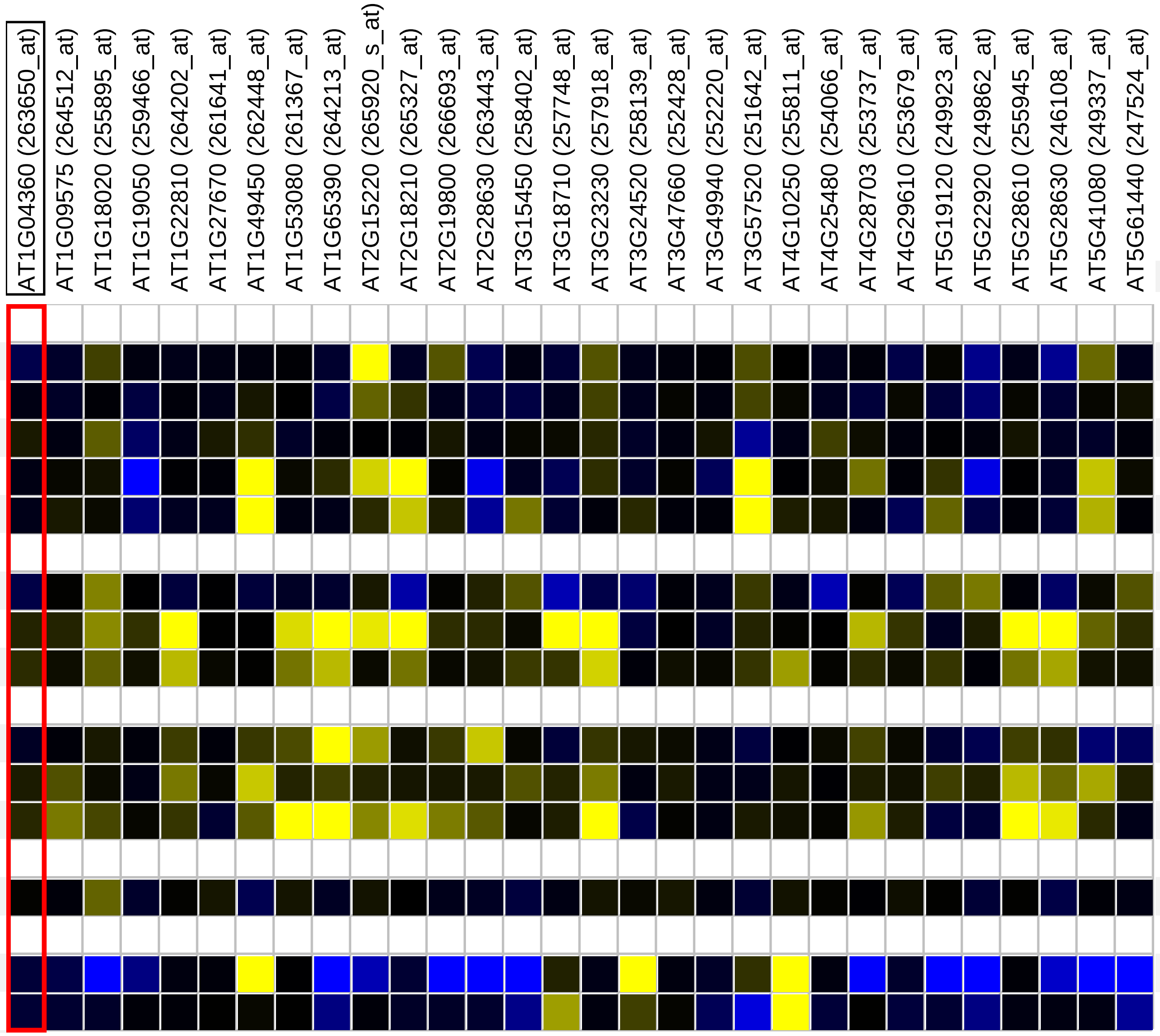

	Log2-ratio	Fold-Change	p-value
		no filter	no filter
▼ Biotic			
A. brassicicola study 3 (Col-0) / mock treated leaf samples (Col-0)	-0.76	-1.80	0.228
B. cinerea / non-infected rosette leaf samples	-0.20	-1.15	0.376
H. arabidopsidis study 4 (Col-0) / untreated seedling samples (Col-0)	0.26	1.19	0.064
P. syringae pv. maculicola (Col-0) / mock treated leaf samples (Col-0)	-0.19	-1.14	0.180
P. syringae pv. tomato study 3 (DC3000) / mock inoculated leaf samples (24h)	-0.25	-1.19	0.017
▼ Chemical			
benzothiadiazole study 3 (Col-0) / untreated (Col-0) plant samples	-0.71	-1.67	0.089
chitin / mock treated seedlings	0.36	1.28	0.015
H2O2 study 3 (Col-0) / untreated seedlings (Col-0)	0.43	1.35	0.005
▼ Elicitor			
EF-Tu (elf18) study 3 (Col-0) / mock treated seedling samples (Col-0)	-0.37	-1.30	0.084
FLG22 (1h) / H2O treated leaf samples (1h)	0.27	1.21	0.036
Pep2 (Col-0) / mock treated seedling samples (Col-0)	0.40	1.32	0.113
▼ Hormone			
salicylic acid / mock treated seedlings	0.04	1.03	0.801
▼ Stress			
heat study 4 / untreated plant samples	-0.57	-1.48	0.043
heat study 11 (Col) / untreated seedling samples (Col)	-0.53	-1.44	0.050

created with GENEVESTIGATOR
