# Supplementary material for: Dual impact of elevated temperature on plant defence and bacterial virulence in Arabidopsis
Source: Nat Commun. 2017 Nov 27;8:1808. doi: 10.1038/s41467-017-01674-2 (PMC5704021; doi:10.1038/s41467-017-01674-2)
Supplement: Supplementary file 9 — Supplementary Data 6 [file 41467_2017_1674_MOESM9_ESM.zip › Genevestigator_RawOutput/Cluster8_1_data.pdf]

Dataset: 19 perturbations (sample selection: SYH)
340 probes (gene selection: SYH_C1_1)

Log2-ratio

-2.5 -2.0 -1.5 -1.0 -0.5 0.0 0.5 1.0 1.5 2.0 2.5

Down-regulated Up-regulated

14 of 14 perturbations fulfilled the filter criteria

Filter values for AT1G01200 (261042_at)

no filter no filter

Arabidopsis thaliana (14)

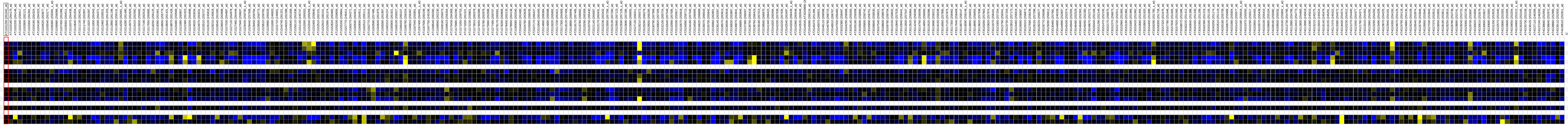

Perturbation	Log2-ratio	Fold-Change	p-value
▼ Biotic			
A. brassicicola study 3 (Col-0) / mock treated leaf samples (Col-0)	-0.76	-1.70	0.044
B. cinerea / non-infected rosette leaf samples	-0.13	-1.09	0.402
H. arabidopsidis study 4 (Col-0) / untreated seedling samples (Col-0)	0.09	1.06	0.582
P. syringae pv. maculicola (Col-0) / mock treated leaf samples (Col-0)	-0.31	-1.24	0.140
P. syringae pv. tomato study 3 (DC3000) / mock inoculated leaf samples (24h)	-0.05	-1.04	0.669
▼ Chemical			
benzothiadiazole study 3 (Col-0) / untreated (Col-0) plant samples	-0.25	-1.19	0.179
chitin / mock treated seedlings	-0.06	-1.04	0.413
H2O2 study 3 (Col-0) / untreated seedlings (Col-0)	0.12	1.08	0.191
▼ Elicitor			
EF-Tu (elf18) study 3 (Col-0) / mock treated seedling samples (Col-0)	0.13	1.10	0.404
FLG22 (1h) / H2O treated leaf samples (1h)	-0.07	-1.04	0.616
Pep2 (Col-0) / mock treated seedling samples (Col-0)	-0.49	-1.40	0.008
▼ Hormone			
salicylic acid / mock treated seedlings	0.17	1.13	0.497
▼ Stress			
heat study 4 / untreated plant samples	0.13	1.10	0.351
heat study 11 (Col) / untreated seedling samples (Col)	-0.05	-1.03	0.753

created with GENEVESTIGATOR
