# Supplementary material for: Dual impact of elevated temperature on plant defence and bacterial virulence in Arabidopsis
Source: Nat Commun. 2017 Nov 27;8:1808. doi: 10.1038/s41467-017-01674-2 (PMC5704021; doi:10.1038/s41467-017-01674-2)
Supplement: Supplementary file 9 — Supplementary Data 6 [file 41467_2017_1674_MOESM9_ESM.zip › Genevestigator_RawOutput/Cluster8_2_data.pdf]

Dataset: 19 perturbations (sample selection: SYH)

101 probes (gene selection: SYH_C1_2)

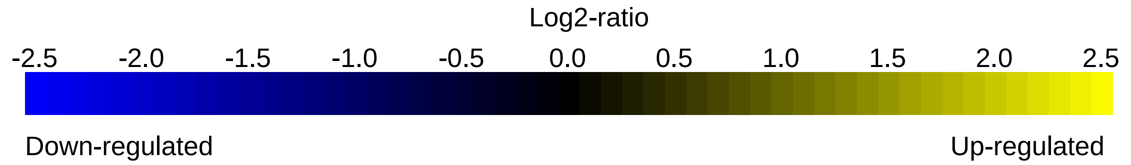

14 of 14 perturbations fulfilled the filter criteria

Filter values for AT5G01015 (251142_at)

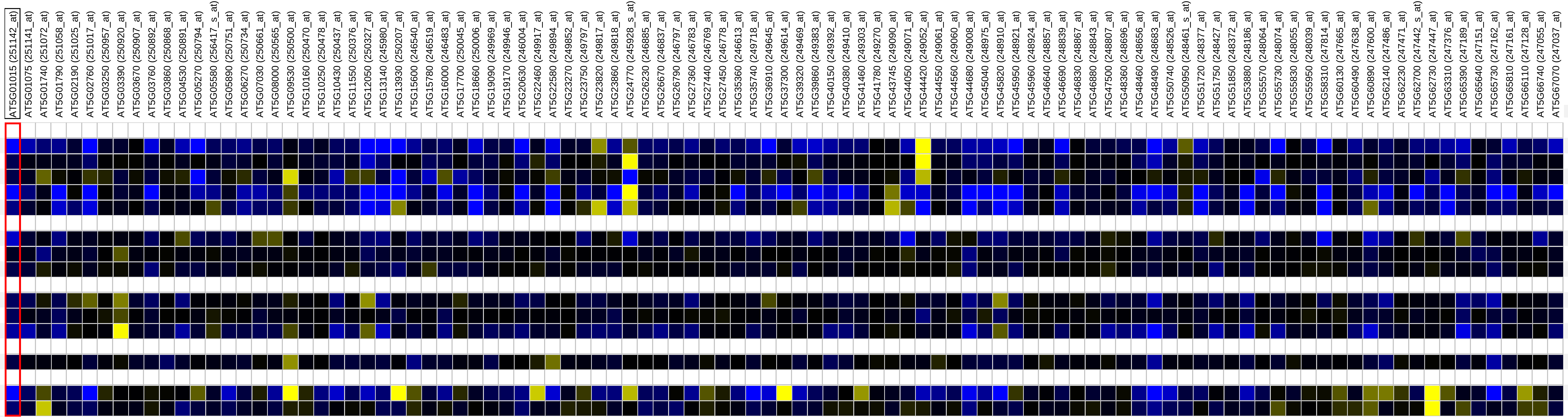

Arabidopsis thaliana (14)	Log2-ratio (no filter)	Fold-Change (no filter)	p-value
▼ Biotic			
A. brassicicola study 3 (Col-0) / mock treated leaf samples (Col-0)	-3.49	-11.55	0.002
B. cinerea / non-infected rosette leaf samples	-0.81	-1.55	0.218
H. arabidopsidis study 4 (Col-0) / untreated seedling samples (Col-0)	-0.74	-1.66	0.104
P. syringae pv. maculicola (Col-0) / mock treated leaf samples (Col-0)	-3.09	-9.04	<0.001
P. syringae pv. tomato study 3 (DC3000) / mock inoculated leaf samples (24h)	-1.30	-2.45	0.010
▼ Chemical			
benzothiadiazole study 3 (Col-0) / untreated (Col-0) plant samples	-2.00	-3.63	0.015
chitin / mock treated seedlings	-0.36	-1.30	0.135
H2O2 study 3 (Col-0) / untreated seedlings (Col-0)	-0.74	-1.67	0.006
▼ Elicitor			
EF-Tu (elf18) study 3 (Col-0) / mock treated seedling samples (Col-0)	-0.94	-1.89	0.014
FLG22 (1h) / H2O treated leaf samples (1h)	-0.89	-1.84	0.005
Pep2 (Col-0) / mock treated seedling samples (Col-0)	-1.58	-2.99	<0.001
▼ Hormone			
salicylic acid / mock treated seedlings	-0.69	-1.62	0.037
▼ Stress			
heat study 4 / untreated plant samples	-2.79	-7.01	0.005
heat study 11 (Col) / untreated seedling samples (Col)	-1.13	-2.11	0.020

created with GENEVESTIGATOR
