# Supplementary material for: Dual impact of elevated temperature on plant defence and bacterial virulence in Arabidopsis
Source: Nat Commun. 2017 Nov 27;8:1808. doi: 10.1038/s41467-017-01674-2 (PMC5704021; doi:10.1038/s41467-017-01674-2)
Supplement: Supplementary file 9 — Supplementary Data 6 [file 41467_2017_1674_MOESM9_ESM.zip › Genevestigator_RawOutput/Cluster9_2_data.pdf]

Dataset: 19 perturbations (sample selection: SYH)

65 probes (gene selection: SYH_C2_2)

Log2-ratio

-2.5 -2.0 -1.5 -1.0 -0.5 0.0 0.5 1.0 1.5 2.0 2.5

Down-regulated — Up-regulated

14 of 14 perturbations fulfilled the filter criteria

Filter values for AT5G18060 (250012_x_at)

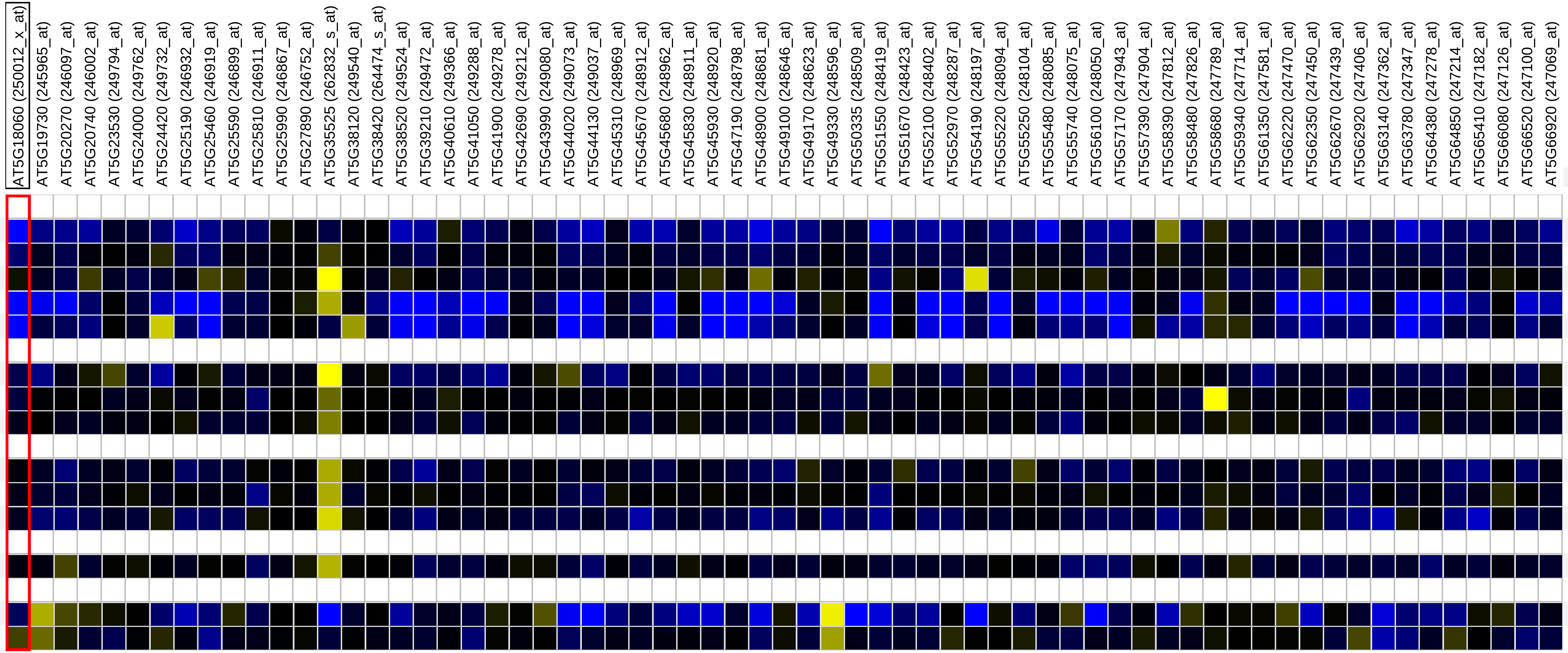

Arabidopsis thaliana (14)	Log2-ratio	Fold-Change	p-value
		no filter	no filter
▼ Biotic			
A. brassicicola study 3 (Col-0) / mock treated leaf samples (Col-0)	-2.69	-6.95	0.018
B. cinerea / non-infected rosette leaf samples	-1.09	-2.15	0.015
H. arabidopsidis study 4 (Col-0) / untreated seedling samples (Col-0)	0.17	1.09	0.719
P. syringae pv. maculicola (Col-0) / mock treated leaf samples (Col-0)	-3.29	-10.22	<0.001
P. syringae pv. tomato study 3 (DC3000) / mock inoculated leaf samples (24h)	-2.58	-5.93	<0.001
▼ Chemical			
benzothiadiazole study 3 (Col-0) / untreated (Col-0) plant samples	-0.75	-1.70	0.041
chitin / mock treated seedlings	-0.61	-1.51	0.012
H2O2 study 3 (Col-0) / untreated seedlings (Col-0)	-0.29	-1.23	0.004
▼ Elicitor			
EF-Tu (elf18) study 3 (Col-0) / mock treated seedling samples (Col-0)	-0.11	-1.07	0.470
FLG22 (1h) / H2O treated leaf samples (1h)	-0.25	-1.22	0.419
Pep2 (Col-0) / mock treated seedling samples (Col-0)	-0.31	-1.22	0.150
▼ Hormone			
salicylic acid / mock treated seedlings	-0.17	-1.12	0.256
▼ Stress			
heat study 4 / untreated plant samples	-0.93	-1.91	0.012
heat study 11 (Col) / untreated seedling samples (Col)	0.64	1.91	0.454

created with GENEVESTIGATOR
