# Supplementary figures and images for: Dual impact of elevated temperature on plant defence and bacterial virulence in Arabidopsis
Source: Nat Commun. 2017 Nov 27;8:1808. doi: 10.1038/s41467-017-01674-2 (PMC5704021; doi:10.1038/s41467-017-01674-2)

115 probes (gene selection: SYH\_C7)

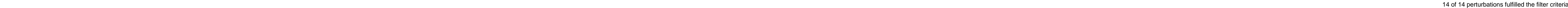

## Arabidopsis thaliana (14)

created with GENEVESTIGATOR

Supplement: Supplementary file 9 — Supplementary Data 6 [file 41467_2017_1674_MOESM9_ESM.zip › Genevestigator_RawOutput/Cluster1_data.pdf]

probes (gene selection: SYH\_C8)

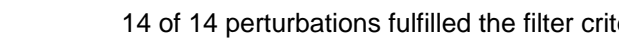

Filter values for 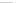 AT1G01480 (259439)

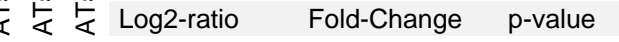

Supplement: Supplementary file 9 — Supplementary Data 6 [file 41467_2017_1674_MOESM9_ESM.zip › Genevestigator_RawOutput/Cluster4_data.pdf]

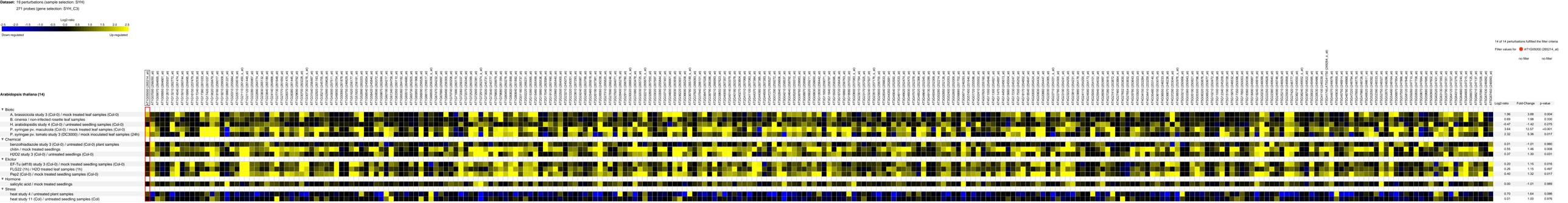

Supplement: Supplementary file 9 — Supplementary Data 6 [file 41467_2017_1674_MOESM9_ESM.zip › Genevestigator_RawOutput/Cluster5_data.pdf]

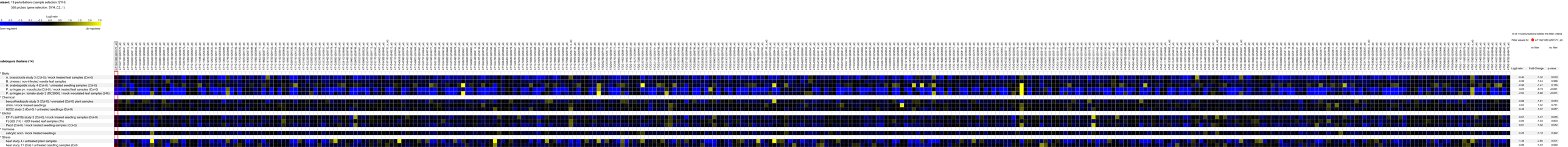

Supplement: Supplementary file 9 — Supplementary Data 6 [file 41467_2017_1674_MOESM9_ESM.zip › Genevestigator_RawOutput/Cluster9_1_data.pdf]
