# Supplementary material for: Dual impact of elevated temperature on plant defence and bacterial virulence in Arabidopsis
Source: Nat Commun. 2017 Nov 27;8:1808. doi: 10.1038/s41467-017-01674-2 (PMC5704021; doi:10.1038/s41467-017-01674-2)
Supplement: Supplementary file 9 — Supplementary Data 6 [file Cluster3_1_data.pdf › Genevestigator_RawOutput/Cluster3_1_data.pdf]

Dataset: 19 perturbations (sample selection: SYH)

348 probes (gene selection: SYH_C0_1)

Log2-ratio

-2.5 -2.0 -1.5 -1.0 -0.5 0.0 0.5 1.0 1.5 2.0 2.5

Down-regulated Up-regulated

14 of 14 perturbations fulfilled the filter criteria

Filter values for ● AT1G02220 (264148_at)

Arabidopsis thaliana (14)

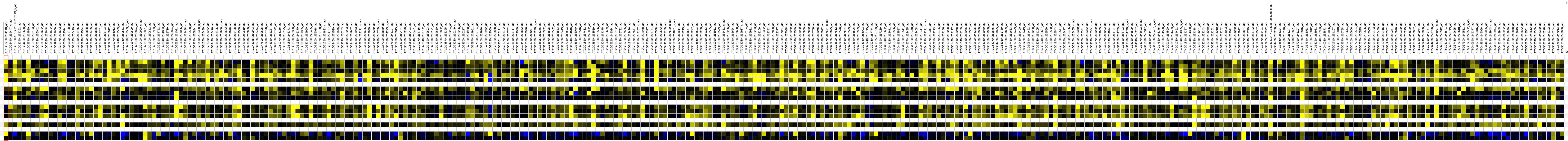

	Log2-ratio (no filter)	Fold-Change (no filter)	p-value
▼ Biotic			
A. brassicicola study 3 (Col-0) / mock treated leaf samples (Col-0)	2.77	5.94	0.031
B. cinerea / non-infected rosette leaf samples	1.57	8.03	0.340
H. arabidopsidis study 4 (Col-0) / untreated seedling samples (Col-0)	0.34	1.27	0.012
P. syringae pv. maculicola (Col-0) / mock treated leaf samples (Col-0)	3.87	14.65	<0.001
P. syringae pv. tomato study 3 (DC3000) / mock inoculated leaf samples (24h)	4.10	17.36	<0.001
▼ Chemical			
benzothiadiazole study 3 (Col-0) / untreated (Col-0) plant samples	0.53	1.45	0.032
chitin / mock treated seedlings	-0.25	-1.14	0.402
H2O2 study 3 (Col-0) / untreated seedlings (Col-0)	-0.25	-1.19	0.077
▼ Elicitor			
EF-Tu (elf18) study 3 (Col-0) / mock treated seedling samples (Col-0)	-0.87	-1.82	0.009
FLG22 (1h) / H2O treated leaf samples (1h)	-0.01	-1.01	0.922
Pep2 (Col-0) / mock treated seedling samples (Col-0)	-0.01	1.00	0.957
▼ Hormone			
salicylic acid / mock treated seedlings	1.57	3.05	0.045
▼ Stress			
heat study 4 / untreated plant samples	4.10	16.57	0.025
heat study 11 (Col) / untreated seedling samples (Col)	0.04	1.03	0.588

created with GENEVESTIGATOR
